# Genetic dissection of *Escherichia coli*'s master diguanylate cyclase DgcE: Role of the N-terminal MASE1 domain and direct signal input from a GTPase partner system

**DOI:** 10.1371/journal.pgen.1008059

**Published:** 2019-04-25

**Authors:** Vanessa Pfiffer, Olga Sarenko, Alexandra Possling, Regine Hengge

**Affiliations:** Institut für Biologie / Mikrobiologie, Humboldt-Universität zu Berlin, Berlin, Germany; Institut Pasteur, CNRS UMR 3525, FRANCE

## Abstract

The ubiquitous second messenger c-di-GMP promotes bacterial biofilm formation by playing diverse roles in the underlying regulatory networks. This is reflected in the multiplicity of diguanylate cyclases (DGC) and phosphodiesterases (PDE) that synthesize and degrade c-di-GMP, respectively, in most bacterial species. One of the 12 DGCs of *Escherichia coli*, DgcE, serves as the top-level trigger for extracellular matrix production during macrocolony biofilm formation. Its multi-domain architecture–a N-terminal membrane-inserted MASE1 domain followed by three PAS, a GGDEF and a degenerate EAL domain–suggested complex signal integration and transmission through DgcE. Genetic dissection of DgcE revealed activating roles for the MASE1 domain and the dimerization-proficient PAS_3_ region, whereas the inhibitory EAL^deg^ domain counteracts the formation of DgcE oligomers. The MASE1 domain is directly targeted by the GTPase RdcA (YjdA), a dimer or oligomer that together with its partner protein RdcB (YjcZ) activates DgcE, probably by aligning and promoting dimerization of the PAS_3_ and GGDEF domains. This activation and RdcA/DgcE interaction depend on GTP hydrolysis by RdcA, suggesting GTP as an inhibitor and the pronounced decrease of the cellular GTP pool during entry into stationary phase, which correlates with DgcE-dependent activation of matrix production, as a possible input signal sensed by RdcA. Furthermore, DgcE exhibits rapid, continuous and processive proteolytic turnover that also depends on the relatively disordered transmembrane MASE1 domain. Overall, our study reveals a novel GTP/c-di-GMP-connecting signaling pathway through the multi-domain DGC DgcE with a dual role for the previously uncharacterized MASE1 signaling domain.

## Author summary

Biofilms represent a multicellular life form of bacteria, in which large numbers of cells live in communities surrounded and protected by a self-generated extracellular polymeric matrix. As biofilms tolerate antibiotics and host immune systems, they are causally associated with chronic infections. Biofilm formation is generally promoted by the ubiquitous bacterial second messenger c-di-GMP. DgcE, one of the 12 diguanylate cyclases that produce c-di-GMP in *E*. *coli*, was previously shown to specifically act as a top level trigger in the regulatory network that drives biofilm matrix production in this bacterium. However, signal input into DgcE itself, which is a large six-domain protein, had remained unknown. Here we demonstrate that DgcE activity is controlled by a novel type of dynamin-like GTPase that directly interacts with the N-terminal membrane-intrinsic MASE1 domain of DgcE. Our finding of a dual function of this MASE1 domain, which is essential for both activation and continuous proteolysis of DgcE, is the first characterization of this widespread bacterial signaling domain. Signal input via the dynamin-like GTPase system suggests that c-di-GMP production by DgcE might be stimulated by the decreasing cellular GTP level during entry into stationary phase, which is precisely the time when biofilm matrix production is turned on.

## Introduction

The discovery and functional analysis of bacterial cyclic dinucleotides, in particular bis-(3´,5´)-cyclic diguanosine monophosphate (c-di-GMP), has opened a new era in the study of second messenger signaling in prokaryotes, revealing a complexity that matches that in eukaryotes [1–4]. C-di-GMP is synthesized by diguanylate cyclases (DGCs, with this activity provided by their GGDEF domains) and degraded by specific phosphodiesterases (PDEs, with either EAL or HD-GYP domains) [5, 6]. These signaling enzymes usually exhibit a modular domain architecture. Diverse N-terminal domains, which in many cases also anchor the enzymes in the cytoplasmic membrane, act as signal input devices responding to often still unknown signals. These DGCs and PDEs antagonistically negotiate levels of c-di-GMP which is bound by a large diversity of effector components. These can be different types of proteins that control various targets by direct interaction or riboswitches in the upstream regions of mRNAs that affect transcriptional elongation or translation [7, 8]. These effector-target systems can simply respond to global changes in the cellular pool of c-di-GMP. However, also local c-di-GMP signaling has been observed, especially in bacterial species that have multiple DGCs and PDEs, some of which can exert surprisingly specific functions based on their direct association with effector/target systems [9–14]. c-di-GMP-controlled targets are involved in fundamentally important cellular and physiological functions, such as the formation of highly antibiotic-tolerant biofilms, motility, virulence, cell cycle progression and development [3, 15–19].

**Fig 1:**
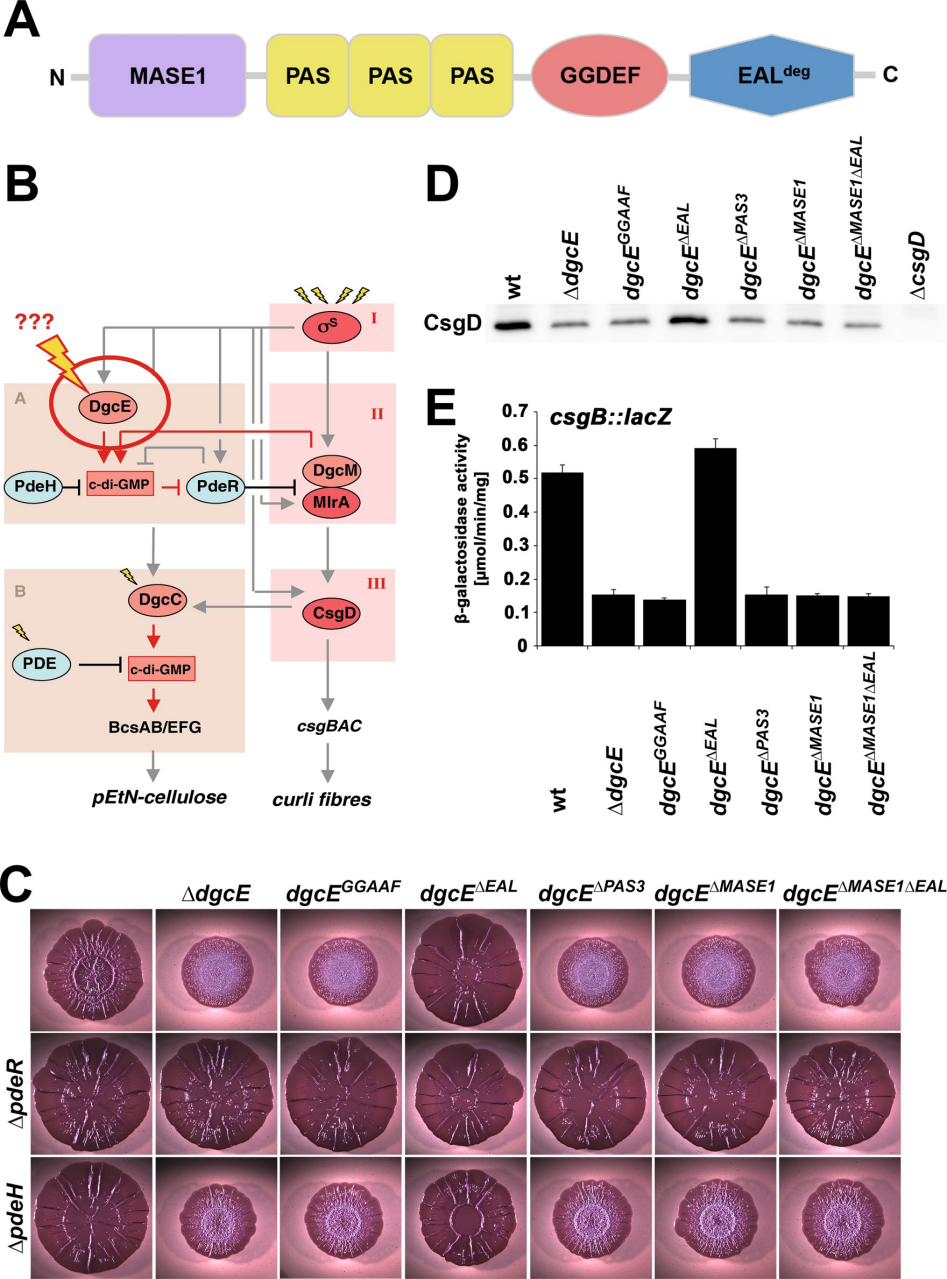
Role of domains in DgcE function. **A:** Domain structure of DgcE. **B:** Regulatory network controlling the production of curli fibres and pEtN-cellulose. The vertical 'backbone' of the network is the transcription factor cascade σ^S^/MlrA/CsgD (in red), with c-di-GMP input occurring via modules A and B, which include the integrated action of different DGCs and PDEs (in light red and light blue, respectively). DgcE is the master DGC at the top level of this hierarchy, with its signal input and processing (indicated by the bolt) being the central topic of this study. **C:** Macrocolonies of AR3110 derivatives with the indicated chromosomal *dgcE* alleles in otherwise wt, *ΔpdeH* and *ΔpdeR* backgrounds were grown on Congo red plates for 5 days at 28°C. **D:** CsgD levels determined by immunoblot analysis in strain AR3110 carrying the indicated chromosomal *dgcE* alleles. Samples were obtained at an OD_578_ of 3.6–3.8, with 6 μg total protein loaded per lane. **E:** *csgB*::*lacZ* expression measured after growth of strain W3110 *Δlac(I-A)* carrying the indicated chromosomal *dgcE* alleles in LB at 28°C for 24 h.

A major c-di-GMP target in *Escherichia coli*–an enteric bacterium existing as a commensal as well as in various types of pathogens–is the production of extracellular matrix polymers during biofilm formation [20]. In macrocolony or pellicle biofilms of *E*. *coli*, these matrix components are amyloid fibres termed curli [21] as well as phosphoethanolamin-modified cellulose (pEtN-cellulose) [22]. Their production occurs in slowly growing cells entering into stationary phase and is under the control of a transcription factor cascade consisting of the stationary phase sigma factor σ^S^ (RpoS), the MerR-like regulator MlrA and the transcription factor CsgD, with the latter directly driving the expression of the curli subunits CsgA and CsgB and indirectly activating cellulose production (summarized in [20]). Positive input by c-di-GMP is required at two positions in this hierarchical control network (see also Fig 1 below): (i) in a circuit involving DgcE, PdeR and DgcM, the target is the activity of MlrA, with the mutually interacting proteins PdeR, DgcM and MlrA representing a case of local c-di-GMP signaling [10, 12], and (ii) further downstream, c-di-GMP is required for directly activating cellulose synthase (BcsAB) [23] as well as the co-localized cellulose modifying system (BcsEFG) [22, 24, 25]. This seems yet another process of local signaling as it requires specifically DgcC, which in turn depends on CsgD for being expressed [26].

A major question mark in this regulatory network is associated with the role of the top level diguanylate cyclase DgcE, which provides for the key trigger that activates the entire cascade thereby leading to CsgD expression and biofilm matrix production. What are the environmental and/or cellular signals that DgcE responds to and how does it do so at the molecular level? With its six-domain architecture (Fig 1A), DgcE is the most complex among the twelve DGCs of *E*. *coli* K-12 [27, 28]. Its N-terminal part consists of a MASE1 domain, a putative sensory domain originally described to have eight transmembrane (TM) segments that also occurs at the N-termini of PDEs and histidine sensor kinases and is found in gamma-, beta- and alpha-proteobacteria as well as in cyanobacteria [29]. The complete hydrophobic N-terminal region in MASE1 domain proteins typically has a total length of about 300 amino acids and–based on hydrophobicity patterns and the distribution of charge (S1 Fig)–contains ten rather than eight transmembrane segments, with this entire region now being annotated as the MASE1 domain. In DgcE this domain is followed by three cytoplasmic PAS/PAC domains, the GGDEF domain and an EAL domain that is degenerate, i.e. it lacks all the key amino acid residues required for c-di-GMP binding and catalysis of the PDE reaction [28].

What is the role of all these domains in signal integration and transduction into and through DgcE? Can an analysis of DgcE lead us to an understanding of the sensory function of the widespread membrane-integral MASE1 domain? Are any other factors involved in signal input into DgcE? To elucidate the molecular functions of DgcE, we focussed on a detailed genetic analysis since DgcE is not only a complex membrane-associated protein, but in the course of our experiments we also discovered a constitutive *in-vivo* turnover of DgcE, i.e. DgcE has properties that efficiently prevent purification and *in-vitro* analysis. This genetic approach allowed us to assign specific roles to the different domains of DgcE and to demonstrate signal input by a GTPase system that directly interacts with the MASE1 domain of DgcE and thereby triggers downstream signaling.

## Results

### DgcE domains contribute differentially to DgcE-dependent activation of the production of CsgD and extracellular matrix

In order to elucidate the function of particular sites and domains in DgcE, we started by introducing specific point and deletion mutations. We chose to do so in the single chromosomal copy of the *dgcE* gene, as DgcE controls a local c-di-GMP signaling pathway, i.e. PdeR-DgcM-MlrA/*csgD*, which involves multiple protein-protein interactions [10, 13] for which stoichiometry of proteins matters. As an experimental readout, we used (i) macrocolony biofilm morphology, which provides for a semi-quantitative assay for curli and pEtN-cellulose production (as further explained in S2 Fig), (ii) direct visualization of cellular CsgD in liquid cultures by immunoblotting, and (iii) a single copy *csgB*::*lacZ* reporter fusion to monitor the activity of the *csgBAC* operon, which encodes the curli subunits.

Two amino acid exchanges (D763A/E764A) in the active site motif of DgcE (GGDEF to GGAAF) resulted in the same strong reduction in macrocolony wrinkling as the complete deletion of *dgcE* (Fig 1C), i.e. a phenotype corresponding to a strong reduction in both curli fibres and pEtN-cellulose [13]. This is also reflected in reduced CsgD levels (Fig 1D) and *csgB*::*lacZ* expression (Fig 1E). Thus, the input of DgcE into the PdeR-DgcM-MlrA/*csgD* pathway relies on its ability to generate c-di-GMP. Although intuitively taken for granted, this has to be clarified, since e.g. the ability of DgcM to support MlrA activity does not require its c-di-GMP synthesis but relies on its direct interaction with MlrA [10].

Internal deletions of the entire transmembrane MASE1 domain or of all three PAS domains (ΔPAS_3_) generated the same null phenotype, i.e. these regions of DgcE play an essential positive role in its signaling into CsgD expression and extracellular matrix production. In order to control for protein expression levels, all the internally deleted variants of DgcE were also generated as Flag-tagged variants (with the tag inserted at the 3´ end of the chromosomal *dgcE* alleles). The presence or absence of the Flag-tag did not affect the macrocolony phenotypes and the Flag-tagged constructs showed that wildtype DgcE and internally deleted DgcE variants were expressed at levels comparable to full size DgcE (see below). In contrast to eliminating the MASE1 and PAS_3_ regions, deleting the C-terminal EAL^deg^ domain resulted in the opposite phenotype, i.e. increased matrix production, which became apparent as even larger, flatter and stiffer macrocolonies that fold into fewer, but higher ridges (Fig 1C, compare also to S2 Fig). A corresponding increase in CsgD levels and *csgB*::*lacZ* was also observed (Fig 1D and 1E). Since the latter assay was done in liquid culture using non-cellulose-producing, but otherwise identical strains (to avoid cell clustering since matrix components are also produced in stationary phase liquid cultures), we also tested the inactivating or activating effects of the various internal deletions in DgcE on macrocolonies of these curli-only producing strains and found them to be the same as in the strains that synthesize both matrix components (S3 Fig). The macrocolony phenotype of the *dgcE*^*ΔEAL*^ strain (Fig 1C) is in fact similar to that of a matrix-overproducing *pdeR* deletion strain (S2 Fig), i.e. the EAL^deg^ domain plays an inhibitory role. This full activation of DgcE^ΔEAL^ could be eliminated by also deleting the MASE1 domain, indicating that the MASE1 domain does not antagonize the inhibitory EAL^deg^ domain, but that its essential activating role is independent of the EAL^deg^ domain.

**Fig 2:**
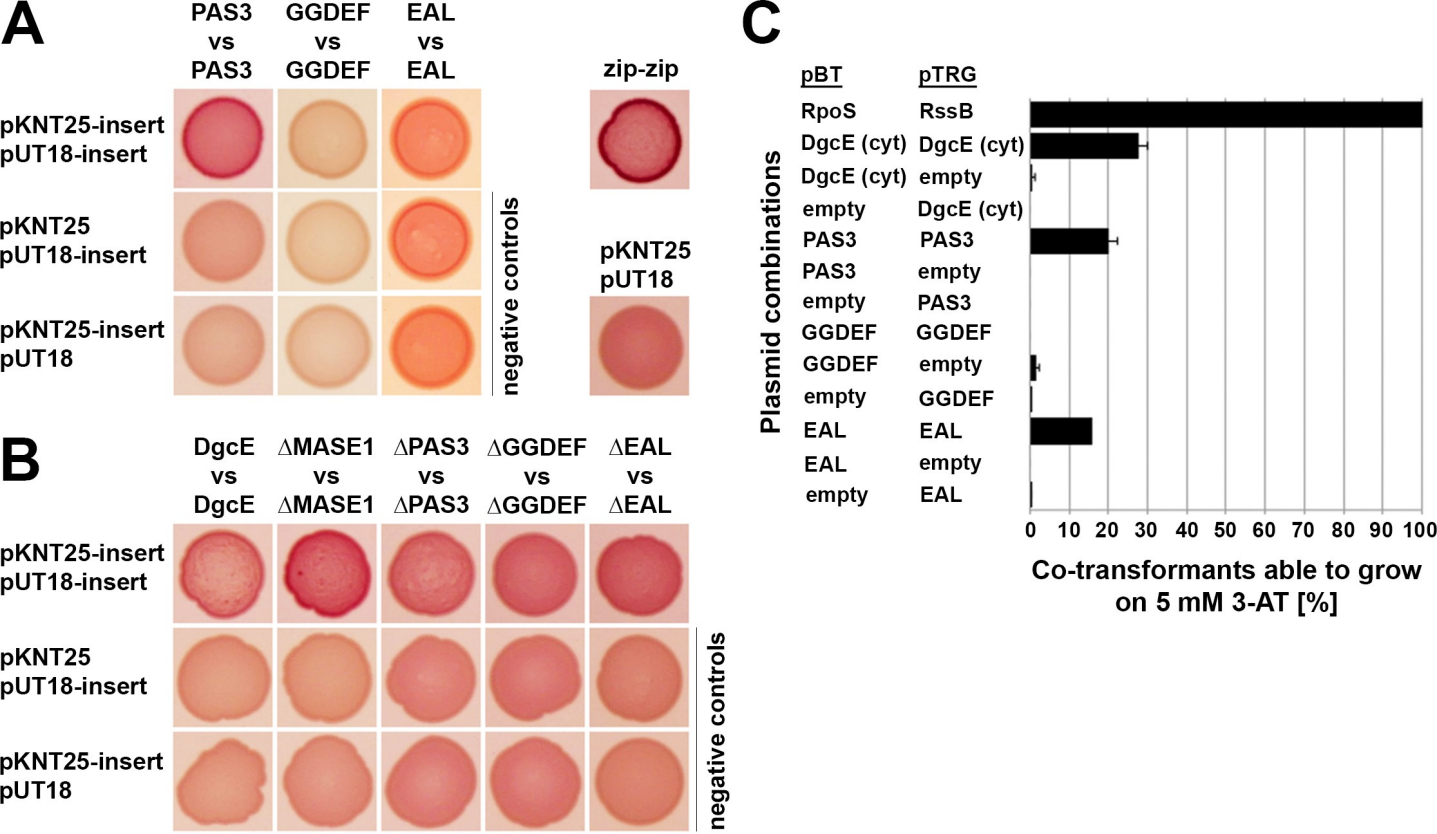
Role of distinct domains in DgcE dimerization. **A:** The isolated region containing all three PAS domains (PAS_3_), as well as the isolated GGDEF and EAL domains of DgcE were tested for dimerization in adenylate cyclase-based two-hybrid assays. **B:** DgcE variants lacking specific domains as indicated were tested by two-hybrid assays. Spotted co-transformants were grown at 28°C for 1 day. As a positive control, the leucin zipper part ('zip') of the yeast GCN4 protein was used. Using pKNT25 and pUT18, the T25 and T18 fragments of adenylate cyclase are attached at the C-termini of the indicated isolated domains and the different DgcE variants. Two-hybrid plasmid vectors used in each combination are listed at the left side of the panels, with 'insert' standing for the indicated proteins cloned into these vectors, vector names alone stand for the empty vectors in control combinations. **C:** The indicated parts of DgcE were tested for dimerisation using an alternative two-hybrid assay (Bacteriomatch; for details, see Materials and Methods). As a positive control, the strongly interacting RpoS and RssB proteins were used.

In a series of epistasis experiments, we combined all these mutations in *dgcE* with full deletion mutations in *pdeR* and in *pdeH*. The macrocolony morphotypes of the double mutants (Fig 1C) confirmed that DgcE acts upstream of PdeR (mutations in *dgcE* had no phenotypic effects in a *pdeR* mutant background), but at the same level as PdeH (all double mutants carrying both the *pdeH* and inactivating deletion mutations in *dgcE* showed an intermediate phenotype, i.e. less wrinkling than *pdeH* alone, but more wrinkling than the corresponding *dgcE* mutations alone; Fig 1C). PdeH is the master PDE that maintains very low global c-di-GMP levels in *E*. *coli*, even under conditions where DgcE is active as a DGC and drives biofilm matrix production [13].

c-di-GMP synthesis depends on the dimerization of DGCs, with each monomer binding one of the two GTP substrate molecules, which allows to generate two phosphodiester bonds in a symmetric manner. DGCs are thus thought to be activated by dimerization promoted by their sensory input or other domains [6]. The isolated GGDEF domains of ten of the twelve DGCs of *E*. *coli* K-12 –including that of DgcE–indeed do not dimerize on their own [13]. This prompted us to test which of the other domains of DgcE can dimerize and may thus promote the dimerization of the GGDEF domain and thereby activation of DgcE. To do so, we used a bacterial two-hybrid system in which an interaction between bait and target proteins reconstitutes a functional two-domain adenylate cyclase (Cya), which allows a *Δcya* mutant of *E*. *coli* to utilize maltose as a carbon source [30, 31]. Testing the isolated domains of DgcE in this system confirmed the inability of the GGDEF domain to dimerize on its own [13], whereas the PAS_3_ region generated a clear dimerization signal (Fig 2A). An apparent dimerization of the isolated EAL^deg^ domain was experimentally not conclusive, since the control assays showed a similar signal. We therefore used a second two-hybrid technology, the Bacterio-Match II system, which is based on transcription initiation of a reporter gene enabled by interacting proteins or domains (which have to be cytoplasmic) fused to lambda cI and α-NTD of RNA polymerase [32]. Here, the entire cytoplasmic region of DgcE (i.e. PAS_3_-GGDEF-EAL^deg^) as well as the isolated PAS_3_ and EAL^deg^ domains, but neither the isolated GGDEF domain nor the control combinations yielded a dimerization signal (Fig 2C). With the MASE1 domain alone, the Cya-based two-hybrid assay could not be performed due to overproduction toxicity, i.e. the combination of the plasmid-encoded MASE1 domain fused to the two adenylate cyclase domains affected growth.

In a complementary 'subtractive' approach, we tested dimerization of DgcE variants lacking distinct domains (Fig 2B). The relatively weak dimerization of full-size DgcE was not significantly reduced by eliminating single domains, consistent with more than one domain being able to promote dimerization (as shown above for the isolated domains). Deleting the MASE1 domain even improved dimerization, possibly because the resulting soluble DgcE^ΔMASE1^ is freely diffusible in the cytosol, which may facilitate dimerization in comparison to the sterically more constrained conditions for the membrane-attached full size DgcE. Overall, these two-hybrid interaction data show that both the activating PAS_3_ as well as the inhibitory EAL^deg^ domain of DgcE have a potential for dimerization.

### Proteolytic turnover of DgcE

**Fig 3:**
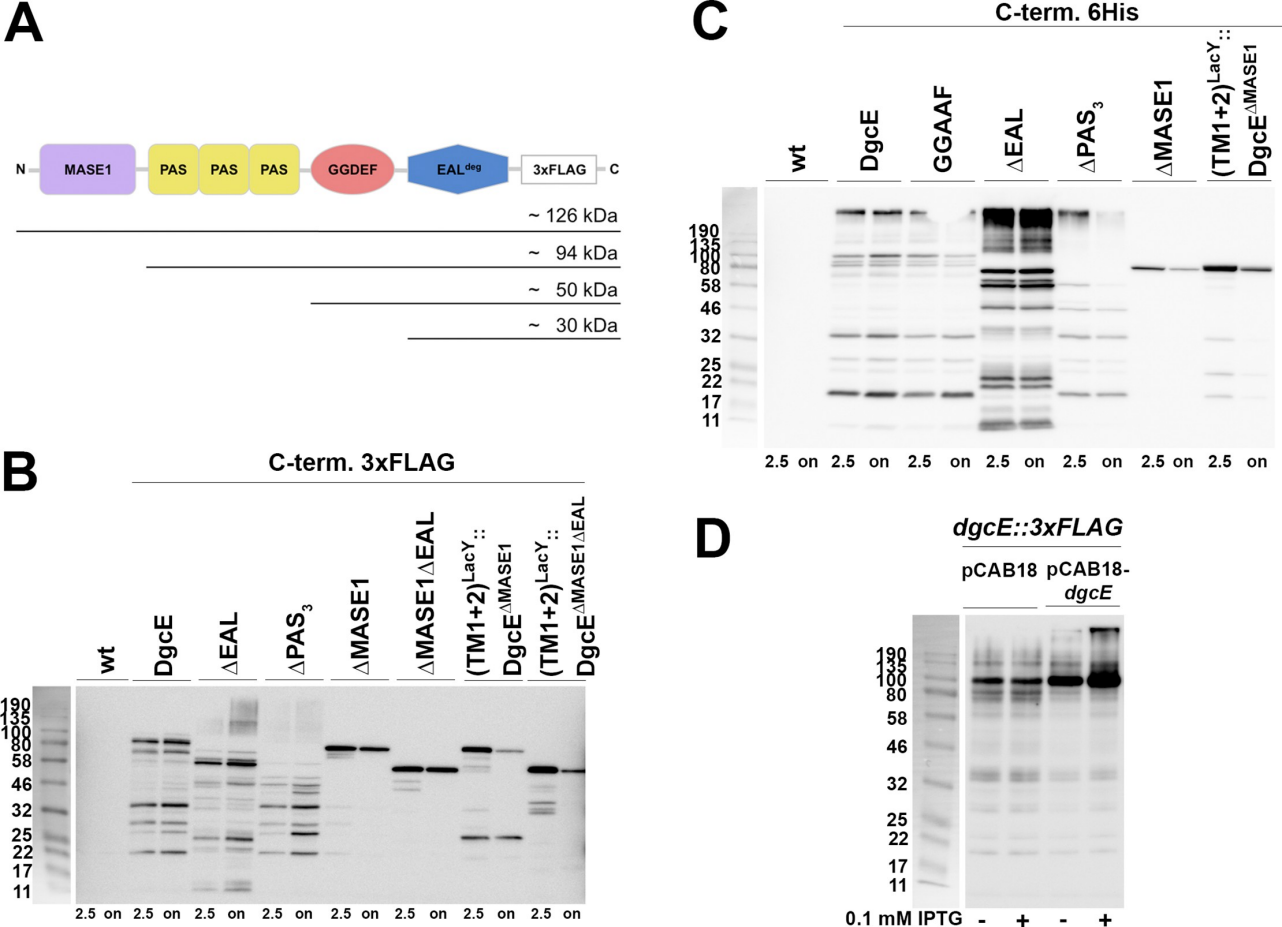
DgcE is subject to proteolytic turnover that proceeds from the N-terminal MASE1 domain to the C-terminal EAL domain. **A:** size of expected proteolytic fragments obtained by degradation starting from the N-terminus of DgcE and pausing between domains. **B:** Immunoblot analysis of chromosomally encoded C-terminally 3xFLAG-tagged DgcE variants in derivatives of strain W3110, with samples taken at an OD_578_ of 2.5 and after overnight growth (on). **C:** Immunoblot analysis performed as in (B), but with the indicated DgcE variants expressed from the low copy number plasmid pCAB18 with a C-terminal 6His tag visualized using anti-6His antibodies. **D:** Immunoblot detecting chromosomally encoded 3xFLAG-tagged DgcE expressed concomitantly with DgcE expressed from plasmid pCAB18 in the absence or presence of the inducer isopropyl-thio-galactoside (IPTG).

**Fig 4:**
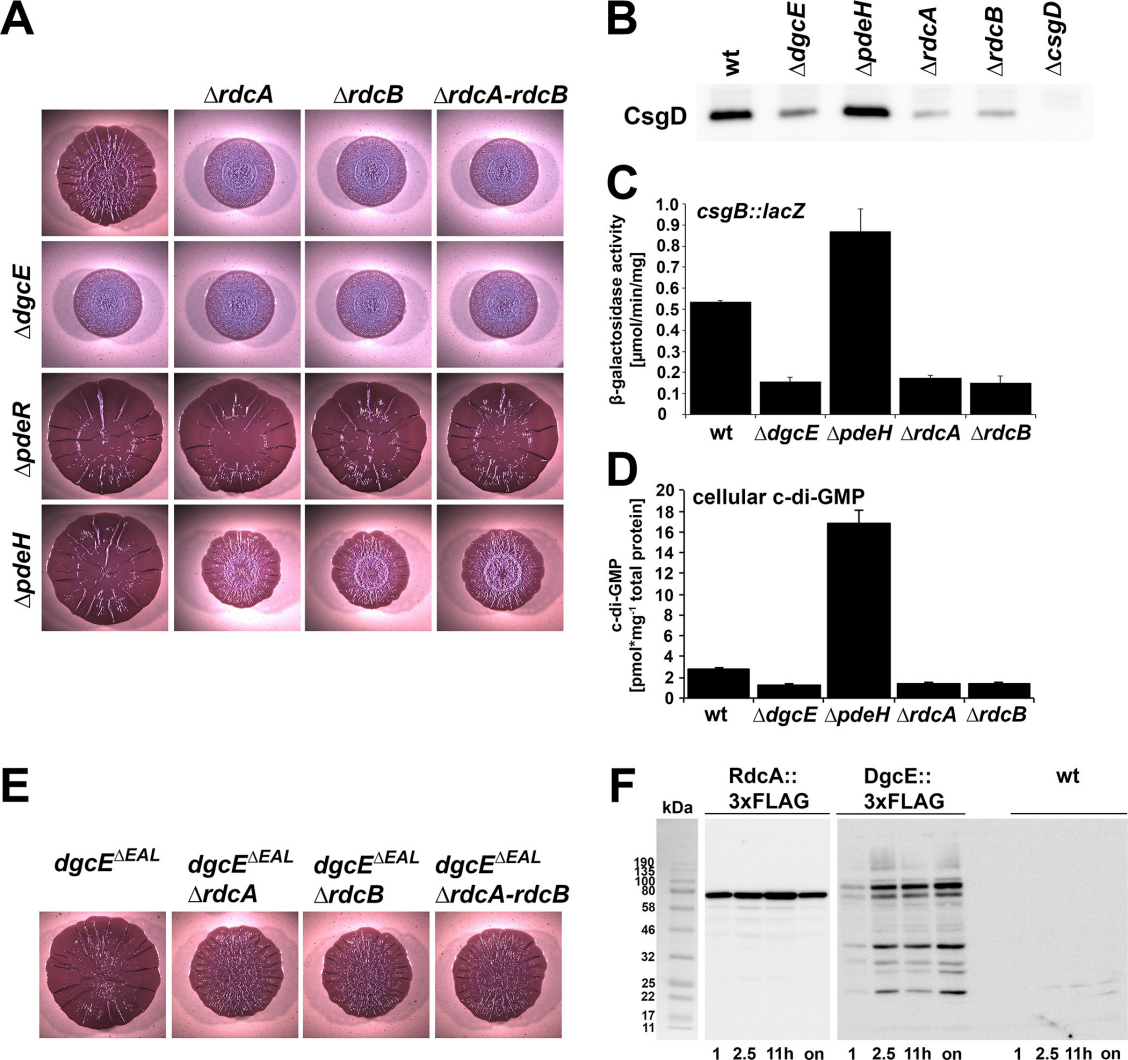
A role for RdcA and RdcB in DgcE-controlled biofilm-associated functions. **A:** Macrocolony morphology of strain AR3110 and its derivatives carrying the indicated single or double mutations. **B:** CsgD levels determined by immunoblot analysis in strain AR3110 carrying the indicated mutations. Samples were obtained at an OD_578_ of 3.6–3.8, with 6 μg total protein loaded per lane. **C:** *csgB*::*lacZ* expression measured after growth of strain W3110 *Δlac(I-A)* carrying the indicated mutations in LB at 28°C for 24 h. **D:** Cellular c-di-GMP concentration determined in strain W3110, sampled at an OD_578_ of 3. **E:** Effects of mutations in *rdcA* and/or *rdcB* on macrocolony morphology of AR3110 derivatives carrying the C-terminally truncated *dgcE*^*ΔEAL*^ allele. **F:** Expression of RdcA and DgcE during the growth cycle. Both proteins carried a chromosomally encoded C-terminal 3xFLAG tag for immunoblot detection, which was performed with samples harvested at the OD_578_ of 1 and 2.5, after 11 h of growth (where cells have reached an OD_578_ of approximately 4.5) and after overnight growth (on). The 3xFLAG-free, but otherwise isogenic strain W3110 (wt) was used as a control.

The observation of different *in-vivo* activities of the various DgcE variants (Fig 1) required to experimentally control for the actual expression of these variants. Using both FLAG-tagged chromosomal variants (as mentioned already above) as well as plasmid-encoded variants carrying a 6His tag, immunoblotting showed overall similar levels of expression. In addition, however, these experiments revealed clear degradation patterns for all variants except for the soluble cytoplasmic DgcE^ΔMASE1^ variant (Fig 3B; for wildtype DgcE, this degradation was already previously noticed by Sarenko et al. (2017)). As the N-terminal transmembrane region of DgcE is required for proteolysis and the tags for visualization are C-terminally located, the ladder-like pattern indicates processive proteolysis from the N- to the C-terminus of DgcE, with the protease pausing or slowing down between domain boundaries as suggested by the sizes of the fragments generated. When we replaced the MASE1 region by the first two transmembrane segments of the lactose carrier LacY (TM1+2^LacY^), the presence of this new membrane-insertion region also allowed degradation (Fig 3B). This suggests that DgcE is attacked by a membrane-associated protease, with the transmembrane MASE1 domain providing for appropriate localization of DgcE rather than for sequence-specific recognition for proteolysis. Proteolysis seems constitutive as it occurred in growing as well as in stationary phase cells (Fig 3B and also Fig 4F below). Since the MASE1 domain is required for activity of DgcE (Fig 1), but it is also the first region to be eliminated by this processive proteolysis, this turnover represents an inactivating mechanism.

Initiation of DgcE proteolysis seems highly efficient since we could not detect chromosomally expressed full-size DgcE protein, i.e. the largest visible band corresponded in size to that of the DgcE^ΔMASE1^ variant (Fig 3A and 3B). Upon overproduction, however, larger bands were detected, in particular an oligomeric form of DgcE, which was not monomerized by SDS treatment during sample preparation and was also larger than expected for a dimer. Interestingly, when DgcE lacked its EAL domain, these oligomers could be observed even when expressed from the chromosomal gene (in stationary phase cells; Fig 3B) and, when plasmid-expressed, they accumulated to higher levels than for wildtype DgcE (Fig 3C). This suggests that the inhibitory function of the EAL domain may involve a destabilization of this oligomeric complex and that this oligomer represents the active form of DgcE. Interestingly, plasmid-encoded DgcE was found to affect *in trans* the chromosomally encoded FLAG-tagged DgcE. In this configuration, the latter accumulated partially as the oligomeric DgcE complex (Fig 3D), i.e. it became better protected against proteolytic attack which most likely reflected a titration of the protease involved.

All this raised the question of the identity of the protease and we therefore tested DgcE turnover in a series of protease knockout mutants (S4 Fig). Processive degradation suggested a general ATP-dependent protease, which either could be itself membrane-associated (such as FtsH) or could be cytoplasmic and may proceed with proteolysis after a membrane-bound protease has generated an initial cut in DgcE. None of the mutations affecting the cytoplasmic ATP-dependent AAA+ proteases ClpXP, ClpAP, Lon or HslUV [33] altered the DgcE degradation pattern. The same was observed for the Lon homolog YcbZ, which lacks ATPase activity. Testing a putative involvement of FtsH was less straightforward since FtsH is essential and its degradation of certain substrates is modulated by the two accessory factors HflK and HflC [34]. However, a *ftsH* deletion is available that is viable due to the presence of a suppressor mutation [35]. When plasmid-encoded DgcE was expressed in strains carrying the *ftsH* deletion and the suppressor or the suppressor alone, it was still degraded (S5 Fig) and also knocking out *hflK* and *hflC* in our standard background did not show any effect (S4 Fig). Furthermore, knocking out HtpX, a membrane-associated protease that is related to FtsH but does not hydrolyze ATP [34], did not alter DgcE proteolysis. Also the rhomboid protease GlpG, which cleaves its substrates within the membrane [34], showed no evidence of being involved in DgcE degradation (S4 Fig). Taken together, these data suggest that DgcE may be degraded either by a very specific still unknown membrane-associated protease or, alternatively, several proteases may be redundantly involved such that knocking out individual proteases alone does not produce a significant effect.

Overall, DgcE is subject to efficient and continuous, i.e. apparently not further regulated, proteolytic turnover by a still unknown protease(s), which proceeds from the membrane-inserted N-terminal MASE1 domain to the C-terminal EAL^deg^ domain of DgcE. This also means that cellular DgcE activity depends on continuous *de-novo* synthesis, i.e. DgcE exhibits a highly dynamic regulation which allows for rapid shut down by proteolysis when expression of DgcE ceases.

### Signal input into DgcE: the Dynamin-like GTPase RdcA (YjdA) and its accessory component RdcB (YjcZ) activate DgcE by direct interaction

The N-terminal MASE1 domain as well as the PAS_3_ region of DgcE are required for DgcE activity and thus may also perceive positive input signals. A mutation in *dgcE* has long been known to partially suppress the motility defect of a *pdeH* mutant [36, 37]. Along with the suppressor in *dgcE* (termed *yegE* at the time), also several other suppressor mutations were described in the study by Girgis et al. (2007). Two of these exactly phenocopied the *dgcE* suppressor mutation and were mapped to *yjdA* and *yjcZ*, which are located in an operon controlled by the flagellar FlhDC/σ^FliA^ transcriptional cascade [36]. This raised the possibility that YjdA and YjcZ might operate in the DgcE pathway, possibly by activating DgcE. Since this turned out to be the case (see below), we propose to rename the two genes as *rdcA* and *rdcB*, respectively (regulators of a diguanylate cyclase; see also Discussion).

Deleting *rdcA* and/or *rdcB* phenocopied a *dgcE* knockout also with respect to macrocolony biofilm formation, with the effects being non-additive, no matter whether combined with deletions of the entire *dgcE* gene (Fig 4A) or with internal deletions in *dgcE* affecting the activating domains only (S6 Fig). Epistasis experiments in double mutants also carrying either *pdeR* or *pdeH* mutations placed the activity of RdcA and RdcB upstream of PdeR, but at the same level as PdeH (Fig 4A), just as shown above for DgcE itself (Fig 1C). Also with respect to CsgD levels (Fig 4B), curli expression as assayed with the *csgB*::*lacZ* reporter fusion (Fig 4C) and cellular c-di-GMP levels (Fig 4D), the *rdcA* and *rdcB* mutations showed precisely the same effects as the *dgcE* mutation.

All these data indicated that RdcA and RdcB activate the production of CsgD, curli and pEtN cellulose and that these proteins act at the level of DgcE. One possibility seemed that they counteract the inhibitory role of the EAL domain of DgcE, but this was excluded by showing that the mutations in *rdcA* and/or *rdcB* still reduced matrix production in macrocolonies of the *dgcE*^*ΔEAL*^ strain (Fig 4E). Thus, the activating RdcA/RdcB system and the inhibitory EAL^deg^ domain affect the output of DgcE independently. The presence or absence of the RdcA/RdcB system also had no effect on the proteolytic turnover of DgcE (S7 Fig). Expression of the *rdcAB* operon–assayed with a FLAG-tagged RdcA expressed from the chromosomal gene–showed expression throughout the growth cycle (Fig 4F), with a minor increase in post-exponential phase, where the expression of flagellar genes is known to transiently increase [38, 39]. This assures co-expression with DgcE, which is further induced in the post-exponential growth phase (Fig 4F) as it is under σ^S^ control [13, 40].

**Fig 5:**
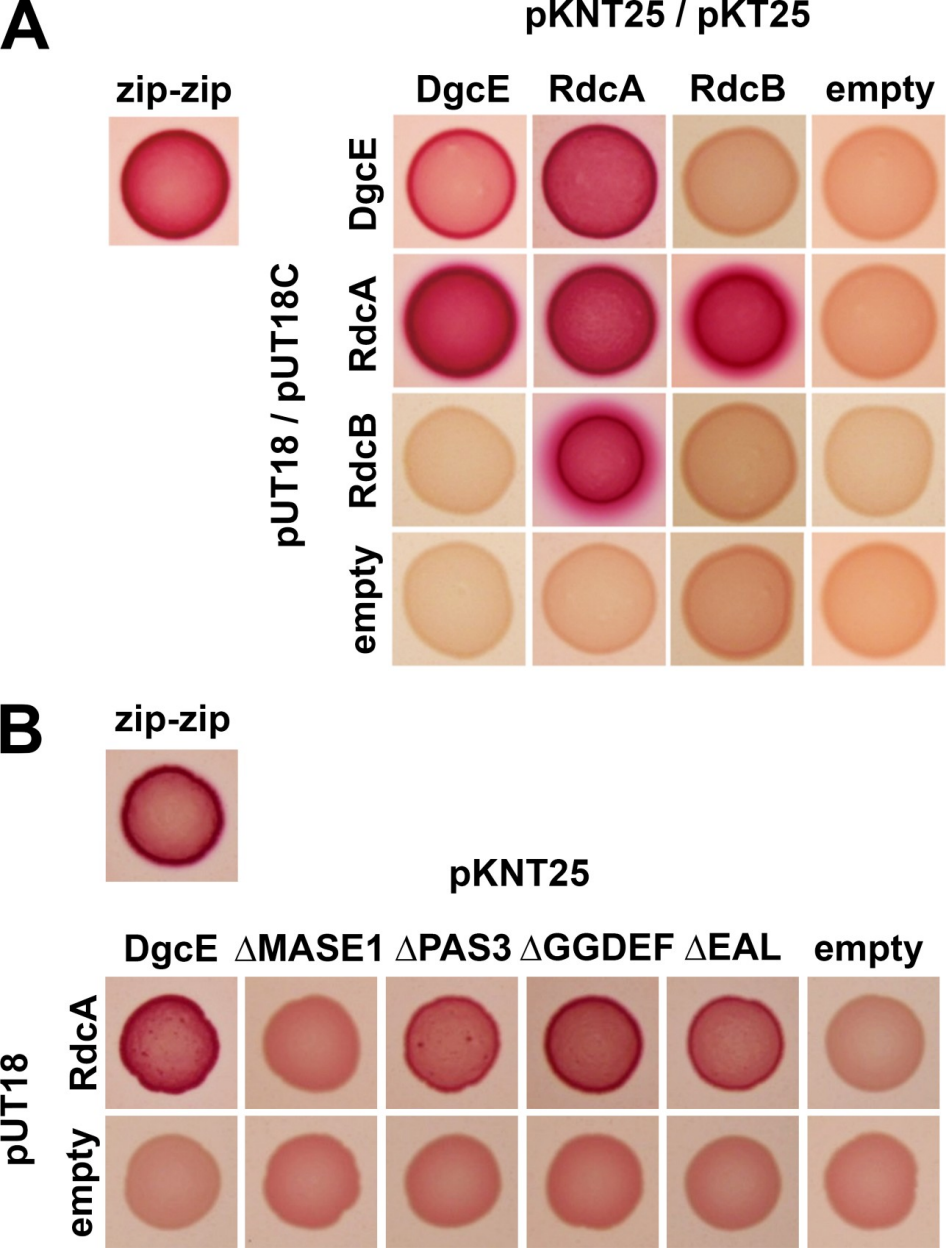
RdcA directly interacts with DgcE and RdcB. **A:** Two-hybrid interaction assays with the indicated full size proteins. All interactions involving DgcE as well as homodimerization of RdcA and RdcB were tested with fusion proteins that carry the T25 or T18 fragments of adenylate cyclase attached at their C-termini (expressed from pKNT25 or pUT18, respectively). The interaction between RdcA and RdcB was tested with fusion proteins that carry T25 and T18 at their N-termini (expressed from pKT25 or pUT18C, respectively), since the corresponding combination with the adenylate cyclase fragments at the C-termini produced heterogeneously colored transformants. **B:** Two-hybrid analysis for interaction between DgcE variants lacking single domains and RdcA. 'empty' stands for the corresponding two-hybrid vector without cloned insert in control combinations.

We then assayed DgcE, RdcA and RdcB for potential direct interactions using the Cya-based bacterial two-hybrid system already described above. RdcA was found to strongly interact with DgcE (Fig 5A). In addition, RdcA could dimerize (or oligomerize) and tightly interacted with RdcB, while RdcB itself did not show any evidence of dimerizing or interacting with DgcE. Two-hybrid testing of RdcA against the series of DgcE variants lacking particular domains, assigned the interaction of RdcA to the N-terminal transmembrane MASE 1 domain of DgcE (Fig 5B).

We conclude that DgcE and RdcA–both with the ability to dimerize–can form a complex, with RdcA also binding to RdcB, indicating that the RdcA/RdcB system tightly cooperates with DgcE. The knockouts of all three genes generate exactly the same downstream effects–including a small but significant reduction in the cellular concentration of c-di-GMP–and RdcA directly targets the N-terminal MASE1 domain of DgcE, which is required for DgcE activity. Taken together, these data indicate that the RdcA/RdcB system activates DgcE to act on the PdeR-DgcM-MlrA pathway which stimulates *csgD* expression.

### The GTPase activity of RdcA is essential for activation of DgcE

How can the RdcA/RdcB system act on DgcE and could this process in turn be controlled by some input signal? While the C-terminal half of RdcA (a 84.4 kDa protein) as well as the smaller RdcB protein (32.9 kDa) do not exhibit any similarities to known protein families, the N-terminal region of RdcA contains a P-loop NTPase region or G domain with similarity to dynamin-like proteins. Dynamins are GTPases [41, 42] and such an activity has also been observed for RdcA in a previous study [43]. The G domain features Walker A and B motifs (also termed G1 and G2 motifs, respectively) involved in GTP binding and hydrolysis, respectively, which is crucial for the diverse functions of dynamin-like proteins [41, 42]. Similar to eukaryotic dynamin, which is involved in clathrin-coated vesicle formation during endocytosis [44], and several prokaryotic GTPases of diverse functions [45], RdcA contains the conserved lysine residue (K82) in its G1 motif (the entire consensus sequence GxxxxGKS is fully conserved in RdcA) and the conserved threonine residue (T103) in its G2 motif. In the dynamin crystal structure the corresponding residues are K44, which is crucial for GTP binding, and T65, which was shown to be part of the active site and to be directly involved in catalysis [46]. Several amino acid replacements were isolated for T65, with the T65D variant of dynamin still binding GTP with the same affinity as the wildtype dynamin, whereas its GTPase activity was >100fold reduced [47].

**Fig 6:**
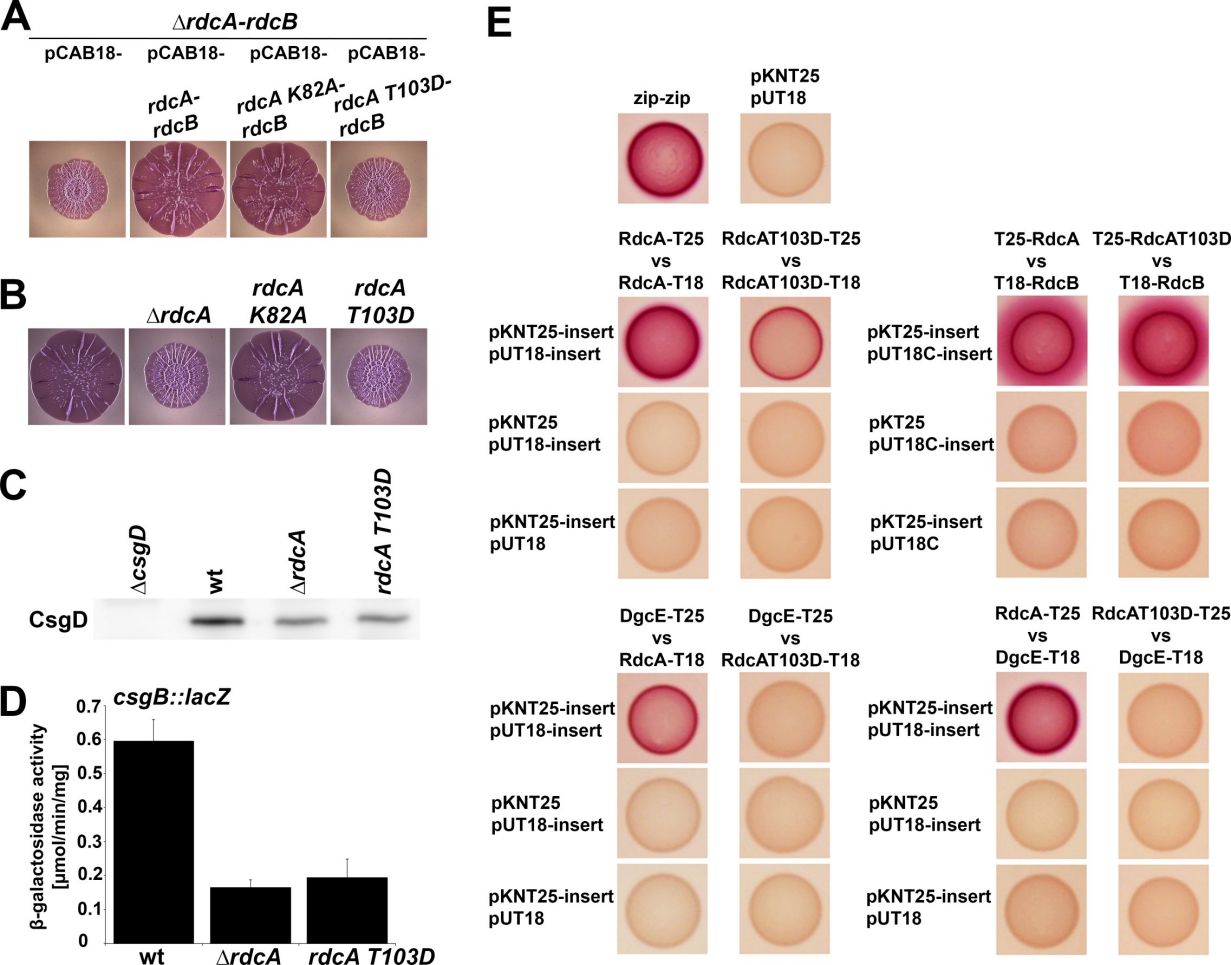
GTPase activity of RdcA is crucial for the activity of the RdcA-DgcE pathway. **A:** Macrocolony phenotypes of a *Δ(rdcA-rdcB)* derivative of strain AR3110 expressing the low copy number plasmid-encoded *rdcA-rdcB* operon containing either wildtype *rdcA*, *rdcA*^*K82A*^ or *rdcA*^*T103D*^. Whereas the plasmid-encoded wildtype *rdcA* and *rdcA*^*K82A*^ alleles show complementation, *rdcA*^*T103D*^ is unable to complement. **B:** Macrocolony phenotypes of strain AR3110 carrying the indicated *rdcA* deletion and point mutations in the chromosome demonstrate that also under conditions of physiological expression, RdcA^K82A^ can support matrix production, whereas RdcA^T103D^ is unable to do so. **C, D:** The chromosomal *rdcA*^*T103D*^ mutation, which eliminates the catalytic threonine required for GTPase activity of RdcA, phenocopies the complete *rdcA* knockout also with respect to the reduction in cellular CsgD levels as detected by immunoblot analysis (**C**) and curli expression as determined using the *csgB*::*lacZ* reporter (**D**). **E:** Two-hybrid interaction assays revealing that the RdcA^T103D^ variant shows reduced dimerization (middle panel, left side) and is unable to interact with DgcE (lower panels), while interaction with RdcB is not affected (middle panel, right side). Two-hybrid plasmid vectors used in each combination are listed at the side of each panel, with 'insert' standing for the indicated proteins cloned into these vectors, vector names alone stand for the empty vectors in control combinations.

In order to test whether GTP binding and GTPase activity are involved in RdcA´s function in the DgcE-controlled pathway, we generated the corresponding amino acid exchanges in RdcA, i.e. K82A and T103D. Plasmids carrying the entire *rdcA-rdcB* operon with these mutations being present in *rdcA* were then tested for complementation of a chromosomal deletion of the *rdcA-rdcB* operon (Fig 6A). The *rdcA*^*K82A*^ allele was found to complement the *rdcA* deletion just as well as the wildtype *rdcA* allele, i.e. RdcA^K82A^ was able to activate DgcE. By contrast, the *rdcA*^*T103D*^ allele was found to be unable to complement. This suggested that GTP per se might not be required for RdcA to activate DgcE, but might rather be inhibitory, with stable GTP binding by RdcA^T103D^ –due to its inability to cleave GTP–'freezing' RdcA in an inactive state unable to activate DgcE. In order to exclude the possibility that these phenotypes were due to plasmid-mediated overexpression of RdcA^K82A^ and/or RdcA^T103D^, we also generated these mutations directly in the chromosomal *rdcA* gene. Also in this single copy configuration, the Walker A mutant version RdcA^K82A^ was fully active, i.e. macrocolony morphology and thus matrix production of the mutant is identical to that of the parental strain (Fig 6B), indicating that GTP binding is dispensible for RdcA-mediated activation of DgcE. By contrast, the chromosomal *rdcA*^*T103D*^ mutant was as defective as the *rdcA* full deletion mutant in terms of macrocolony morphology (Fig 6B), the cellular CsgD level (Fig 6C) and curli expression as tested by the *csgB*::*lacZ* reporter fusion (Fig 6D). In order to exclude that these phenotypes might be caused by a lack of expression, we also Flag-tagged both the wildtype *rdcA* and the *rdcA*^*T103D*^ alleles in the chromosome. The RdcA^T103D^ variant was indeed expressed just like the wildtype RdcA protein (S8A Fig). Furthermore, the two Flag-tagged strains produced the same macrocolony phenotypes (S8B Fig) as the corresponding non-Flag-tagged strains (compare to Fig 6B). Thus, GTPase activity is essential for RdcA to exert its activating function in DgcE-mediated signaling.

This raised the question whether the GTPase-defective RdcA^T103D^ variant could be altered in its direct interaction with DgcE. As shown by two-hybrid analyses, interaction patterns of RdcA were indeed strongly affected by the T103D mutation. Not only was its ability to dimerize (or oligomerize) reduced, but its interaction with DgcE was completely abolished (Fig 6E). By contrast, the T103D mutation in RdcA had no effect at all on the ability of RdcA to interact with RdcB, which also confirmed that the T103D mutation does not alter the overall structure of RdcA. In conclusion, these data support the concept that GTP hydrolysis by RdcA is crucial to allow its direct interaction with DgcE and therefore for its ability to activate the DgcE-triggered downstream pathway that stimulates the production of CsgD and therefore the synthesis of the biofilm matrix components curli and pEtN cellulose.

## Discussion

### DgcE as a prototype of a multi-domain enzyme in c-di-GMP signaling

With its MASE1-PAS_3_-GGDEF-EAL domain architecture, DgcE is a prototype of c-di-GMP signaling enzymes in more than one respects. On the one hand, the function of the membrane-intrinsic putative sensory MASE1 domain, which also occurs e.g. in two-component histidine kinases [29] has remained unknown; on the other, DgcE is a representative of a large subgroup of c-di-GMP signaling enzymes with a PAS-GGDEF-EAL domain architecture. The latter usually have either GGDEF domain-mediated DGC activity or EAL domain-dependent PDE activity, with the respective other domain being degenerate and in some cases exerting a regulatory function. Already the very first DGCs and PDEs identified in *Komatagaeibacter xylinus* (formerly *Acetobacter xylinum*) belonged to this group of PAS-GGDEF-EAL proteins [48]. Since then numerous such enzymes have been found in various bacteria, e.g. the PDEs RbdA and RmcA in *Pseudomonas aeruginosa* [49, 50], PdeB in *Shewanella oneidensis* [51] and the already mentioned trigger PDE PdeR in *E*. *coli* [10, 12]. DgcE clearly is a DGC, since its GGDEF motif (the active site in DGCs) is required for its output function (Fig 1C) and essentially all specific residues that in EAL domains are involved in c-di-GMP and/or metal binding as well as in catalysis [52, 53] are not conserved in the degenerate EAL domain (EAL^deg^) of DgcE, which indicated a regulatory role for this domain. In contrast to many soluble cytoplasmic PAS-GGDEF-EAL domain proteins, DgcE is membrane-attached, suggesting a complex signal transduction that involves both the additional N-terminal transmembrane region, i.e. the MASE1 domain, and the PAS_3_ region.

The top position of DgcE in the hierarchical network (Fig 1B) that controls the production of biofilm matrix components, i.e. amyloid curli fibres and pEtN cellulose, also pointed to a role as a key trigger of this network [10, 37]. The well-studied output, i.e. matrix production, provided us with clear phenotypes and other experimental readouts for studying this function of DgcE. However, we also discovered an efficient and continuous turnover of DgcE, which does not seem to involve a specific proteolytic recognition site in DgcE nor a single specific protease that could be knocked out. As a consequence, this efficient degradation of DgcE could not be experimentally eliminated and therefore purification and biochemical analyses of full-size DgcE were unfeasible, i.e. our study had to built on genetics and *in-vivo* analyses.

### A role for the PAS_3_ region in dimerization and activation of DgcE

PAS domains (named after the prototypical proteins Per, Arnt and Sim) are ubiquitous in all kingdoms of life and, despite high sequence divergence, exhibit similar structure [54, 55]. Their ability to bind diverse small molecules and cofactors makes them prominent sensor domains with a potential to react for instance to oxygen, redox changes or light. In bacteria they are found in many signaling proteins such as histidine kinases, chemotaxis receptor proteins and c-di-GMP signaling enzymes. PAS domains often act as mediators of the dimerization of their downstream domains, which explains the prominence of the PAS-GGDEF arrangement, as DGC activation requires dimerization of the GGDEF domain [6]. Our data indicate that this is also the role of the PAS domains in DgcE, since the PAS_3_ region is both required for DgcE activity (Fig 1) and can dimerize or oligomerize (Fig 2). The finding that also the RdcA/RdcB system as well as the N-terminal transmembrane MASE1 domain of DgcE–which interacts with RdcA–are essential for activation of DgcE (as discussed below), suggests that the PAS_3_ region is important for signal transmission rather than for initial signal perception. Nevertheless, additional and possibly differential signal input via the three PAS domains, perhaps by sensing oxygen or cellular redox signals, is not excluded and will have to be clarified in future structural studies.

### The inhibitory EAL^deg^ domain destabilizes DgcE oligomers

Deleting the C-terminal EAL^deg^ domain of DgcE stimulated the output activity of the DgcE pathway (Fig 1), i.e. this domain performs an inhibitory function. Since it is degenerate in the crucial amino acid positions involved in c-di-GMP binding and catalysis [52, 53], this inhibition cannot be due to degradation of the c-di-GMP generated by the adjacent GGDEF domain of DgcE. EAL domains in general can form dimers [3] and this is so for the EAL^deg^ domain of DgcE as well (Fig 2). Importantly, we observed higher order complexes of the DgcE variant that lacks the EAL^deg^ domain (Fig 3). These complexes were clearly larger than dimers and may be tetramers, which are quite common among DGCs [6]. Since DgcE^ΔEAL^ is also highly active, these oligomers may represent the active form of DgcE, i.e. include the productive GGDEF domain dimers. In addition, despite identical transcriptional and translational control, DgcE^ΔEAL^ was always present at higher levels than wildtype or other variants of DgcE, indicating a lower rate of degradation. As proteolysis initiates from the N-terminus of DgcE, the C-terminal EAL^deg^ domain is unlikely to promote proteolysis directly. Rather, the EAL^deg^ domain may do so indirectly by interfering with the formation of the larger protein complex, which could afford some protection against proteolytic attack–actually a common mechanism to control proteolysis [56].

Taking together all these evidences, an attractive hypothesis to explain the inhibitory role of the EAL domain in DgcE could be that this domain disturbs the functionally productive oligomerization of DgcE, possibly by dimerizing itself in a sterically interfering manner. By contrast, productive oligomerization would be promoted by the interaction of the MASE1 domain of DgcE with RdcA/RdcB and dimerization of the PAS_3_ region which stimulates GGDEF domain dimerization. In this scenario, DgcE would not just show simple activation, but it rather seems the balance between antagonistic negative and positive mechanisms–all based on various interactions of its domains–that would determine the actual regulatory outcome.

### Dual function of the MASE1-containing transmembrane domain in DgcE proteolysis and activation

As large N-terminal transmembrane MASE1 domains were found at the N-termini of c-di-GMP signaling enzymes as well as histidine sensor kinases [29], they obviously are somehow involved in signal input but their actual molecular function had remained enigmatic. Here we show that the MASE1 domain of DgcE plays crucial roles in membrane localization, activity and proteolytic turnover of DgcE.

In DgcE, the MASE1 domain is crucial for proteolytic turnover–which proceeds from the N-terminus to the C-terminus of DgcE–since its deletion resulted in stabilization of DgcE (Fig 3). However, proteolytic turnover was still observed, when the MASE1 domain was replaced by the first two transmembrane segments of the lactose carrier LacY. Thus, DgcE proteolysis requires membrane localization indicating the involvement of a membrane-localized protease(s). Moreover, the MASE1 domain of DgcE does not contain a specific 'degron', i.e. a proteolytic recognition motif [56, 57], but may be recognized due to a partially unfolded structure. The MASE1 domain features a striking number of glycine and proline residues in its transmembrane segments (S1 Fig), i.e. alpha helix-disrupting amino acids that usually are underrepresented in functional transmembrane proteins. This should result in a relatively disordered structure [29], that is likely to be prone to recognition by quality control proteases. Similarly, the first two transmembrane segments of LacY are probably disordered when deprived of the additional transmembrane segments present in the native structure of the whole carrier protein. In *E*. *coli*, MASE1 domains are also present in two c-di-GMP-specific PDEs, PdeA (YfgA) and PdeF (YfgF), which have a MASE1-GGDEF^deg^-EAL domain architecture. In immunoblots, PdeA generates a proteolytic degradation pattern similar to that of DgcE [13]. PdeF is expressed only under anaerobic conditions [58], but ectopic expression under aerobic conditions also results in protein bands on immunoblots that are smaller than expected for the full-size protein [59]. Thus, MASE1 domains may generally confer proteolytic turnover to membrane-associated signaling proteins that carry such domains at their N-termini.

Taken together, all this points to one or more protein quality control proteases that should be generally conserved, membrane-associated and operating in a processive manner on DgcE. Based on these criteria, the most likely protease candidates seem FtsH and HtpX, two related membrane-bound protein quality control proteases that use HflK, HflC and QmcA as accessory factors [60]. While none of the single gene knockouts in this system stabilized DgcE (S4 Fig and S5 Fig), FtsH and HtpX are known to operate in a substrate-overlapping redundant manner, with HtpX becoming essential in a *ftsH* mutant background, which does not allow to generate double mutants [35, 60, 61]. Instead of recognizing specific amino acid motifs, this system initiates proteolysis upon detecting partially unfolded regions in target membrane proteins [60]. Among the ATP-driven general proteases, FtsH also generates the lowest force in unfolding substrate proteins [62] and HtpX even lacks ATPase activity [60], which would be consistent with the pausing of processive proteolysis between the stably folded DgcE domains that generates the observed proteolytic 'ladders' (Fig 3). Initiation of proteolysis at the MASE1 domain, however, seems highly efficient, since the full size protein was hardly detectable on immunoblots, when expressed at the normal rate from the chromosomal copy of the *dgcE* gene. Overall, our data suggest that two or even more proteases may operate efficiently on DgcE in a redundant manner. This also means that DgcE is subject to a highly dynamic control, which allows for rapid shut-off if required, since ongoing synthesis of DgcE is required for its activity in controlling CsgD and matrix production.

Besides its role in proteolysis, the MASE1 domain is also crucial for the function of DgcE in controlling CsgD and biofilm matrix production (Fig 1) as the MASE1 domain provides the site of interaction between DgcE and the activating GTPase RdcA (Fig 5B; as discussed further below). These two functions of the MASE1 domain seem to be independent from each other (apart from proteolysis limiting DgcE activation by RdcA in time), since the presence or absence of RdcA does not affect the turnover of DgcE (S7 Fig), which seems to proceed continuously. Thus, both activation and proteolytic inactivation of DgcE crucially depend on its MASE1 domain. Moreover, this raises the possibility that N-terminal MASE1 domains in other signaling proteins may also not only promote degradation but could also serve as a docking site for additional proteins that provide for signal input.

### RdcA/RdcB–a GTPase system that activates the DgcE pathway to control CsgD and biofilm matrix production

In the case of DgcE, the signaling protein that conditionally docks onto the MASE1 domain, is RdcA, which in turn binds the RdcB protein. RdcA consists of an N-terminal dynamin-like GTPase domain followed by an uncharacterized C-terminal region of about equal size, but in contrast to classical membrane-associated dynamins [44], RdcA is a soluble cytoplasmic protein. The smaller and soluble RdcB protein shows no similarities to proteins of known function. More than ten years ago, mutations in these genes (named *yjdA* and *yjcZ* at the time), which belong to the large FlhDC-controlled flagellar regulon, were found to suppress the motility defect of a *pdeH* mutant, just as a mutation in *dgcE* (*yegE*) did [36]. This provided for a rationale for investigating a putative role of these proteins in DgcE-mediated c-di-GMP signaling, which is crucial to turn down motility and induce biofilm functions during entry into stationary phase [37, 63]. In parallel to our study, a role in submerged biofilms in a microfluidic device and interactions between DgcE, YjdA and YjcZ were also found independently by other researchers [64]. Furthermore, YjdA was also proposed to be involved in chromosome segregation as it was found to interact *in vitro* with clamp protein (DnaN), i.e. a protein with a key role at the replication fork. Therefore, CrfC (colocalization at the replication fork with clamp) was proposed as a new name for YjdA [43]. Unfortunately, however, most of the *in-vivo* experiments reported in this study designed to show a function in DNA replication were done with either YjdA or fluorescent reporters expressed from plasmids. Notably, its interaction with DnaN was also not affected by the presence or absence of GTP [43]. In addition, knocking out *yjdA* has no effect on the growth rate, which clearly excludes a vital mechanistic role in the essential processes of DNA replication and segregation. Thus, it seems more likely that YjdA may play some uncharacterized regulatory role in DNA replication and thus possibly represents a 'moonlighting‘ enzyme, i.e. a protein recruited by evolution to serve in two independent functional contexts [65]. By contrast, knocking out YjdA generates a clear DgcE-mediated biofilm-related phenotype, which depends on its GTP binding/GTPase status and also requires YjcZ, i.e. the second gene product of the *yjdA-yjcZ* operon. Thus, we consider its function in DgcE-dependent c-di-GMP signaling the major function of this system, for which we therefore propose RdcA and RdcB as novel names (for regulators of a diguanylate cyclase; this also opens the possibility of a systematic nomenclature for additional DGC regulators to be identified in the future).

**Fig 7:**
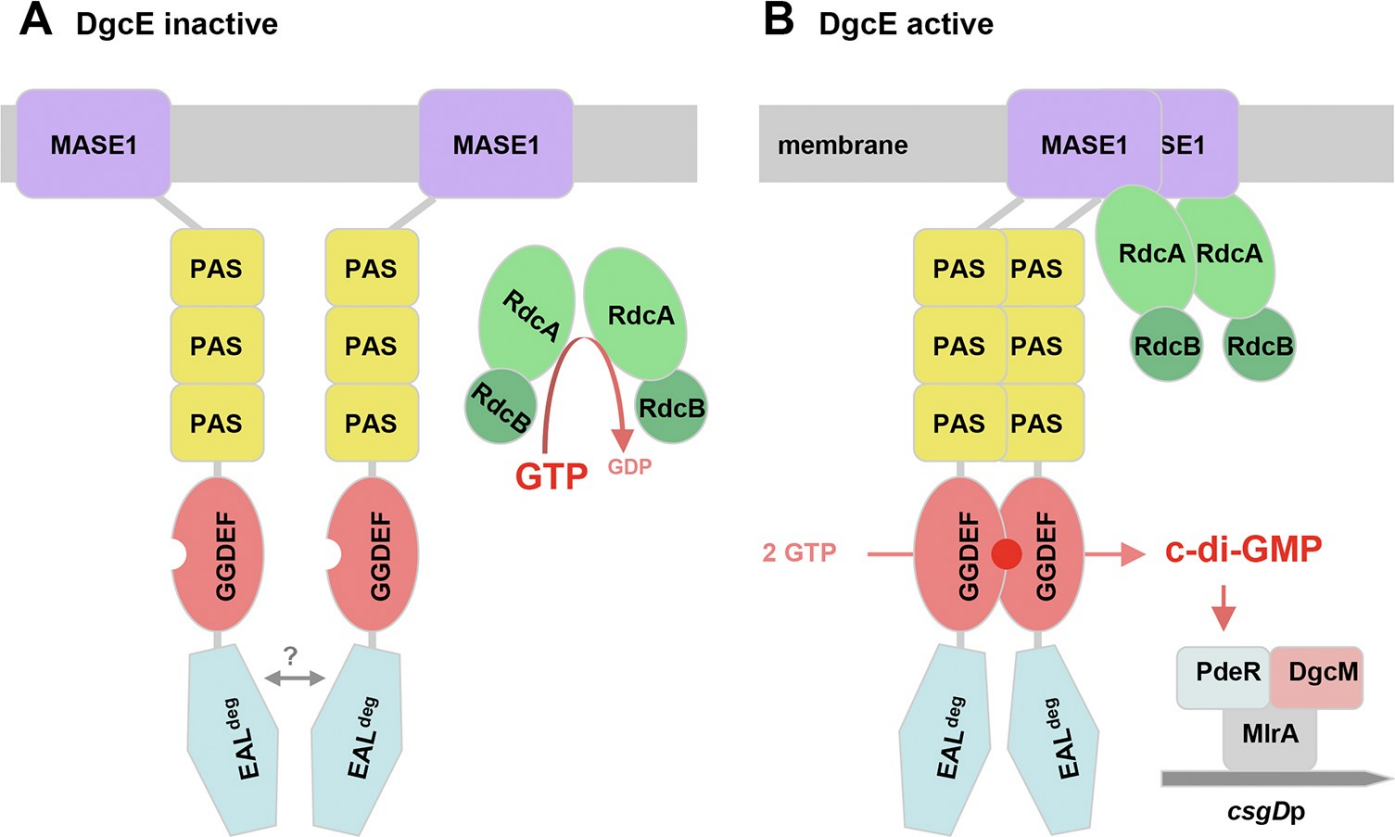
Model of the control of DgcE activity by the GTPase RdcA and its accessory factor RdcB. **A:** At high cellular GTP levels, the RdcA/RdcB complex is predominantly in the GTP-bound form that is unable to interact with the MASE1 domain of DgcE, whose GGDEF domain remains in the monomeric and enzymatically inactive form since the C-terminal EAL domain counteracts dimerization via the PAS_3_ region. **B:** During entry into stationary phase, the cellular GTP level is decreased substantially, the RdcA/RdcB complex is predominantly in the nucleotide-free form that binds to the MASE1 domains of two DgcE molecules, which promotes an alignment of the PAS_3_ and GGDEF domains, thereby overcoming the anti-oligomerizing effect of the EAL domain of DgcE. For simplicity, the c-di-GMP production-promoting forms of both the RdcA/RdcB complex as well as DgcE are depicted here as dimers, but these may also form higher order oligomers (as suggested by immunoblot data in Fig 3). C-di-GMP produced by the GGDEF domains is bound and degraded by the trigger PDE PdeR, thereby relieving inhibition imposed by PdeR onto DgcM and the transcription factor MlrA, which results in the initiation of *csgD* transcription by the DgcM/MlrA complex.

As shown here, RdcA and RdcB are both required for DgcE to stimulate–via the PdeR-DgcM-MlrA pathway–the expression of the biofilm regulator CsgD and thus the production of curli fibres and pEtN-cellulose (Fig 4). RdcA, which is able to dimerize and also binds RdcB, interacts specifically with the N-terminal MASE1 domain of DgcE (Fig 5), i.e. the putative sensory input domain, which is essential for the output activity of DgcE (Fig 1). Thus, the RdcA/RdcB system seems to activate DgcE by direct interaction, with the RdcA dimer (or oligomer) possibly bringing together two DgcE molecules and thereby promoting the alignment and dimerization of the PAS_3_ and GGDEF domains of DgcE, which allows the two GGDEF domains to synthesize c-di-GMP (Fig 7). For this activating function of RdcA, its ability to hydrolyze GTP is essential (Fig 6). This was shown with the RdcA^T103D^ variant, which lacks the essential catalytic threonine residue in the G2 region that is highly conserved in dynamin-like GTPases. RdcA^T103D^ still interacted with its partner protein RdcB *in vivo*, but its ability to dimerize (or oligomerize) was reduced and its interaction with DgcE was completely abolished (Fig 6E).

This could mean that GTP hydrolysis might provide a "powerstroke" for a large conformational change of RdcA that in turn may affect its quaternary structure and thus activation of DgcE in a manner similar to the function of more canonical dynamin-like proteins in driving membrane restructuring events [45, 66–68]. Alternatively, the GTP-bound form of RdcA might be unable to activate DgcE because GTP may act as an allosteric inhibitor. Our data obtained with the RdcA^K82A^ variant (Fig 6A and 6B) are clearly in favor of this second scenario. The K82A mutation eliminates the conserved lysine residue in the Walker A/P-loop motif of dynamin-like protein that is essential for GTP binding [42, 69]. Our finding that this *rdcA*^*K82A*^ allele is fully functional–no matter whether expressed in single copy from the chromosome or from a low copy number plasmid–strongly indicates that the DgcE-activating form of RdcA is the nucleotide-free protein and that GTPase activity is required to rid RdcA of GTP, which acts as an inhibitor.

In contrast to other types of GTPases, dynamin-like GTPases have low affinities for GTP [42, 47]. Since the cellular GTP level is around 1 mM in rapidly growing *E*. *coli* cells and drops five-to-tenfold during transition into stationary phase [70, 71], an inhibitory role of GTP–preventing RdcA from interacting with DgcE (Fig 6E)–could serve as a sensory input into the system. Thus, the system may be fine-tuned in a way that the cellular fraction of RdcA in a non-GTP-bound and therefore DgcE-activating state may increase during entry into stationary phase. Notably, both the RdcA/RdcB system as well as DgcE are present in cells already during earlier phases of the growth cycle (Fig 4F), but their activation–detectable as the beginning of CsgD expression–occurs during the period of entry into stationary phase [37, 72], i.e. coincides with the decrease in the cellular GTP pool during this phase of the growth cycle. Future studies of the mechanistic role of RdcB as a co-activator for RdcA, which could act as a GTPase-activating factor or as a nucleotide release factor, will shed more light on these mechanistic details.

### Conclusion and perspectives

The importance of DgcE as the 'master diguanylate cyclase' for turning down motility and switching on the production of biofilm matrix components is matched by its functional complexity as already suggested by its six domains, which make DgcE one of the largest proteins in *E*. *coli*. Taken together, DgcE seems to make use of the entire tool box of molecular control, including (i) small molecule signaling, with GTP exerting a dual function as a potential sensory input as well as a substrate for generating the output molecule c-di-GMP, (ii) a balance of antagonistic regulatory protein-protein interactions rather than simple activation, (iii) oligomerization in the active state and (iv) proteolysis as a counteracting inactivating process. The integration of all these mechanisms allows for highly dynamic and fine-tuned output control.

Overall, our study also presents a framework for future studies of this key node in the regulatory network that controls *E*. *coli* biofilm formation. Thus, the molecular details and structural consequences of GTP binding and hydrolysis by RdcA, the molecular function of RdcB as a regulatory factor for RdcA and the regulated interaction between RdcA and the MASE1 domain of DgcE will have to be worked out in future genetic and biochemical studies. Also whether and how DgcE and possibly other signaling proteins with N-terminal MASE1 domains are degraded by redundantly acting quality control proteases should be explored further. Finally, DgcE also interacts with its downstream target, the trigger PDE PdeR, and–despite the key role of DgcE-synthesized c-di-GMP in triggering this trigger–has little, if any effect on the constantly very low cellular c-di-GMP pool [13]. Thus, the molecular details of this apparently local downstream signaling by DgcE also deserve further investigation.

## Materials and methods

### Bacterial strains

The strains used are derivatives of the *E*. *coli* K-12 strains W3110 [73] or AR3110, which is a direct derivative of W3110, in which codon 6 (the stop codon TAG) in the chromosomal copy of *bcsQ* was changed to the sense codon TTG [74]. Knockout mutations in *dgcE*, *pdeH*, *pdeR*, *csgD*, *clpP*, *clpA*, *clpX* and *lon* are full open reading frame deletions/antibiotic resistance cassette insertions previously described [10, 37, 40, 74, 75]. The newly constructed mutant alleles *glpG*::*kan*, *hflC*::*kan*, *hflK*::*kan*, *hslVU*::*kan*, *htpX*::*kan*, *qmcA*::*kan*, *rdcA*::*kan*. *rdcB*::*kan*, *rdcA-rdcB*::*kan* and *ycbZ*::*kan* are deletion/insertion mutations generated by one-step inactivation [76] using the oligonucleotide primers listed in S1 Table. When required, cassettes were flipped out using the protocol of Datsenko and Wanner [76]. Mutations were transferred using P1 transduction [77]. Strains carrying the single copy *csgB*::*lacZ* reporter fusion also carry a Δ(*lacI-A*)::*scar* deletion as previously described [13, 75]. In order to test effects of knocking out FtsH protease, a derivative of W3110 carrying *ΔftsH3*::*kan sfhC21 zad220*::Tn*10* as well as an otherwise isogenic *ftsH*^+^ strain carrying only the suppressor (*sfhC21*) and the co-transducible *zad220*::Tn*10* were used [78].

Chromosomally encoded C-terminally 3xFLAG-tagged constructs were generated using plasmid pSUB11 as a PCR template and the oligonucleotide primers listed in S1 Table following a procedure based on lambda-RED technology [79], with wildtype *dgcE*::*3xFlAG* being previously described [13].

### Bacterial growth conditions

Cells were grown in liquid LB medium under aeration at 28 or 37°C. Antibiotics were added as recommended [77]. Liquid culture growth was followed as optical density at 578 nm (OD_578_).

Growth of macrocolony biofilms was previously described [74, 80]. Briefly, 5 μl of the overnight cultures (free of extracellular matrix, since grown in liquid LB at 37°C) were spotted on salt-free LB agar plates supplemented with Congo red and Coomassie brilliant blue (40 μg/ml and 20 μg/ml, respectively; referred to as 'Congo red plates') for the detection of Congo-Red binding (indicative of curli and cellulose production). For growing plasmid-containing strains, 100 μg ml-1 ampicillin was included in the agar plates. In order to achieve reproducible colony morphology, the volume and water content of the agar-containing medium has to be precisely controlled, i.e. plates have to be prepared under exactly identical conditions. All macrocolonies that had to be compared in a given experiment were grown on a single agar plate. Since cellulose and curli fibre expression occurs only below 30°C in *E*. *coli* K-12 strains, cultures were grown at 28°C.

### Mutagenesis of *dgcE* and *rdcA*

All oligonucleotide primers used for mutagenesis are listed in S1 Table. All mutated alleles were verified by PCR and DNA sequencing.

Point mutations within *dgcE* (*dgcE*^*GGAAF*^) and *rdcA* (*rdcA*^*K82A*^, *rdcA*^*T103D*^) were generated using a four-primer/two-step PCR protocol [81]. Two primers carried the mutation and were complementary to each other (mutagenic primers, MP, in S1 Table), whereas the other two primers bound up- and downstream of the region of the mutation. In a first step, two separate PCR fragments were generated (primer combinations A and B) that overlapped at the region of the mutation and covered either the up- or downstream region. In the second step, these fragments were mixed together as DNA templates for a PCR reaction using the up- and downstream primers only in order to generate the final PCR product. This fragment contained the mutated gene region flanked by up- and downstream regions necessary for homologous recombination into the chromosome (see below).

For generating *dgcE* alleles with deletions eliminating internal DgcE domains (*dgcE*^*ΔPAS3*^, *dgcE*^*ΔGGDEF*^) the reverse MP primer were used that bind directly upstream of the domain sequence that needed to be deleted and were combined with a forward primer binding further upstream of that domain. The forward MP primer contained a sequence that was complementary to the reverse MP primer plus a ~20 nt sequence which bound directly downstream of the domain sequence that needed to be deleted. By combining the forward MP primer with a further downstream binding reverse primer, a PCR product was generated, that contained the nucleotide sequence up- and downstream of the domain that needed to be deleted. After the second step, in which both PCR products were used together as DNA-templates for a PCR reaction using the up- and downstream primers only, the final DNA fragment lacking the single domain was generated and could be used for homologous recombination.

For generating the *dgcE*^*ΔMASE1*^ and *dgcE*^*ΔEAL*^ alleles, two-primer combinations were sufficient. *dgcE*^*ΔMASE1*^ was generated by a forward mutagenic primer containing an upstream region and the start of *dgcE* fused to a site directly downstream of the transmembrane (TM) domain-encoding region and a reverse primer that binds further downstream in the *dgcE* gene. *dgcE*^*ΔEAL*^ was generated by a reverse MP primer containing the DNA sequence directly downstream of the *dgcE* sequence fused to a ~ 20 nt segment directly upstream of the region encoding the EAL domain and a forward primer that binds further upstream in the *dgcE* gene.

When generating mutated *dgcE* alleles for cloning into a vector, the whole *dgcE* gene containing the mutation was amplified carrying appropriate restriction sites.

### Introduction of point mutations into the chromosome

For introducing the *dgcE*^*GGAAF*^, *rdcA*^*K82A*^ and *rdcA*^*T103D*^ alleles as well as deletion alleles lacking the coding regions for single DgcE domains into the chromosomal background, a two-step method related to the one-step-inactivation protocol [76] was applied. All oligonucleotides for this procedure are listed in the S1 Table. The procedure uses a fragment of plasmid pKD45 [76], encoding a kanamycin-resistance cassette and the *ccdB* toxin cassette under the control of a rhamnose-inducible promoter, which is first introduced into the target locus in the chromosome (step 1), followed by its recombinatorial replacement using a PCR fragment with the desired allele (step 2), which is selected for by growth in the presence of rhamnose [81]. For generating the (*TM1+2)*^*LacY*^::*dgcE*^*ΔTM*^ mutant allele, a longer synthetic DNA fragment (obtained from GeneArt® Strings, Invitrogen) was used (for sequence, see S1 Table). All other *dgcE* mutant alleles, *rdcA*^*K82A*^ and *rdcA*^*T103D*^ were generated using a four-primer/two-step PCR protocol (see above). Correct allelic states of the resulting transformants were verified by PCR and DNA sequencing.

### Construction of plasmids

Plasmids were constructed using oligonucleotide primers listed in S1 Table. The relevant sequences of the generated plasmids were verified by PCR and DNA sequencing. Unless otherwise indicated, genomic DNA from the *E*. *coli* K12 strain W3110 was used as a template for PCR.

For *in-vivo* two-hybrid assays, relevant genes were cloned into pKT25/pKNT25 as well as into pUT18/pUT18C (Euromedex), which generates hybrid proteins with fragments of *B*. *pertussis* adenylate cyclase fused to the N-termini/C-termini of relevant proteins. For cloning *dgcE*^*ΔPAS3*^ and *rdcA*^*T103D*^, DNA fragments generated by PCR from the respective chromosomal mutant strains were used. Cloning of *dgcE*^*ΔGGDEF*^ was performed by a four-primer protocol (see above for mutagenesis).

For complementation and overexpression assays performed with mutant derivatives of the *E*. *coli* K12 strains AR3110 and W3110, *dgcE* alleles, *rdcA-rdcB*, *rdcA*^*K82A*^*-rdcB* and *rdcA*^*T103D*^*-rdcB* were cloned into the low copy number *lacI*^*q*^ *tac* promoter vector pCAB18 [38]. C-terminal 6His tag codons fused to *dgcE* alleles were introduced via the oligonucleotide primers. For cloning *dgcE*^*ΔPAS*^::*6His*, *dgcE*^*GGAAF*^::*6His*, *rdcA*^*K82A*^*-rdcB* and *rdcA*^*T103D*^*-rdcB* into pCAB18 genomic DNA from the respective mutant strains was used (see above for mutagenesis).

### Bacterial two-hybrid analysis for testing protein-protein interactions *in vivo*

Protein-protein interactions were generally assayed using an adenylate cyclase-based bacterial two-hybrid system [30]. The construction of the hybrid plasmids from pUT18/pUT18C and pKT25/pKNT25 vectors is described above. Plasmids were co-transformed into a Δ*cya* derivative of the K12 strain W3110 and incubated for 24 h at 28°C on MacConkey agar plates supplemented with maltose (1%), ampicillin (100 μg/ml) and kanamycin (50 μg/ml). Single colonies were resuspended in 50 μl sterile water, 5 μl of this suspension was spotted onto fresh MacConkey agar plates and incubated at 28°C for up to 2 d. All colonies to be compared were grown together on a single agar plate, but separate close-up photographs were taken to obtain higher photographic resolution. Red colonies indicate utilization of maltose which depends on cAMP which indicates reconstitution of adenylate cyclase via direct interaction of the proteins fused to the otherwise separate adenylate cyclase domains. The stably dimerizing leucin zipper part ('zip') domain of the yeast transcription factor GCN4 (cloned onto pKT25 and pUT18C) was used as a positive control [30].

For testing interactions between soluble proteins or domains, also the Bacterio-Match II two-hybrid system (Agilent Technologies) was used, in which candidate proteins or domains are linked to the NTD of lambda cI (expressed from pBT) and to the bacterial RNA polymerase alpha-NTD (expressed from pTRG), with co-expression of interacting proteins leading to expression of the *HIS3* gene. This suppresses histidin auxotrophy of the *E*. *coli* reporter strain (a derivative of XL1-Blue MRF’) in a manner that can be fine-tuned by adding the His3 inhibitor 3-amino-1,2,4-triazole (3-AT) [32]. The detailed procedure as well as the two-hybrid plasmids expressing hybrid proteins containing different DgcE domains have been described previously [13].

### SDS polyacrylamide gelelectrophoresis and immunoblot experiments

For immunoblot analyses of 3xFLAG- and 6His-tagged proteins, samples were taken at different time points during growth in LB medium. For CsgD analysis samples were taken at an OD_578_ of 3,5–4 (CsgD expression starts at an OD_578_ of 2.5 and is shut off again later in stationary phase; [72]). Samples corresponding up to 100 μg of total cellular protein were re-suspended in SDS-PAGE sample buffer and incubated for 10 min at 70°C and 15 min at 100°C. Samples were adjusted such that all samples to be compared contained similar amounts of total protein. These were then loaded along with a protein size marker and run on SDS-polyacrylamid (10%) gels. Proteins were detected by immunoblotting as previously described (Lange and Hengge-Aronis, 1994) using antibodies against CsgD (custom-made by Pineda Antikörper Service; used at 1:10000 dilution), 6His-tag (Bethyl Laboratories, Inc.) or 3xFLAG-tag (Sigma). Anti-rabbit or anti-mouse IgG horseradish-peroxidase conjugate from donkey (GE Healthcare) was used (at 1:20000 dilution) for protein visualization in the presence of Western Lightning® Plus-ECL enhanced chemiluminescence substrate (PerkinElmer). Use of the WesternC Precision Plus Marker (Biorad) required also the addition of Streptactin-HRP conjugate. Non-specific protein bands were identified by comparison of the band patterns of recombinant strain samples with those of wildtype *E*. *coli* K-12 samples with similar amounts of cellular protein loaded on the same gels.

### Determination of cellular c-di-GMP levels

Strains were grown at 28°C under aeration in LB medium. At an OD_578_ of 3, 10 ml culture volume was pelleted (4°C, 5000 rpm, 30 min) and stored at -80°C. Sample extraction and analysis of c-di-GMP by LC-MS/MS was performed as described previously [82]. Intracellular concentrations of c-di-GMP were calculated by using the standard OD/cell mass ratio [77]. Extractions were performed in biological triplicates.

### Determination of β-galactosidase activity

β-galactosidase activity was assayed by use of *o*-nitrophenyl-β-D-galactopyranoside (ONPG) as a substrate and is reported as mmol of *o*-nitrophenol per min per mg of cellular protein [77]. Experiments showing the averaged expression of *lacZ* fusions were done in three or more biological replicates.

### Stereomicroscopy of macrocolony biofilms

Macrocolonies were visualized at 10-fold magnification with a Stemi 2000-C stereomicroscope (Zeiss; Oberkochen, Germany). Digital photographs were taken with an AxioCam ICC3 digital camera coupled to the stereomicroscope, which was operated using AxioVision 4.8 software (Zeiss).

## Supporting information

S1 Fig MASE1 domain proteins. **A:** Sequence of the MASE1 domain of DgcE. Hydrophobic transmembrane segments are highlighted in grey, hydrophilic loop regions on the cytoplasmic and periplasmic sides of the inner membrane are overlined in blue and red, respectively. Color code of amino acids: blue, positively charged side chains; red: negatively charged side chains, dark yellow: the helix-breaking amino acids proline and glycine. **B:** Hydropathy plots for five proteins with N-terminal MASE1 domains that were selected to represent different clades of bacteria (*E*. *coli*: γ-proteobacteria; *C*. *crescentus*: α-proteobacteria; *R*. *solanacearum*: β-proteobacteria; *Synechocystis*: cyanobacteria). In all cases the MASE1 domain (highlighted in red) is discernable as a predominantly hydrophobic domain of approximately 290–300 amino acids with a characteristic hydrophobicity pattern indicative of ten transmembrane segments. A window length of 11 amino acids was used for generating the hydropathy plots [83].
(TIFF)

S2 Fig Roles of the biofilm matrix, i.e. amyloid curli fibres and pEtN-cellulose in macrocolony morphology. Macrocolonies of the *E*. *coli* K-12 strains AR3110 and the indicated mutant derivatives, which produce either curli or cellulose or no matrix component at all, were grown on Congo red plates for 5 d at 28°C. The *pdeR* knockout mutation produces higher levels of both matrix components, which results in even larger, flatter and stiffer macrocolonies, which buckle up in fewer but higher radial ridges.
(TIF)

S3 Fig Flag-tagging chromosomal alleles of *dgcE* does not affect macrocolony phenotypes and thus extracellular matrix production. Macrocolonies of the *E*. *coli* K-12 strains W3110, which produce curli fibres but no pEtN cellulose, and the indicated chromosomal *dgcE* mutant derivatives (with the Flag tag sequence inserted at the 3'-end of *dgcE*) were grown on Congo red plates for 5 d at 28°C.
(TIF)

S4 Fig Mutations in various known protease genes do not affect the proteolytic pattern of DgcE. Immunoblot analysis of chromosomally encoded C-terminally 3xFLAG-tagged DgcE was performed with derivatives of strain W3110 carrying the indicated chromosomal deletion mutations, with samples taken after overnight growth in LB at 28°C.
(TIF)

S5 Fig Proteolysis of DgcE is not abolished in a *ftsH* knockout mutant. Viability of a *ftsH* mutant requires the presence of a specific suppressor [35]. Immunoblot analysis of plasmid-encoded C-terminally 6His-tagged DgcE was performed with the strain carrying the suppressor alone (contr. 1 and 2) or the *ΔftsH* and suppressor mutations in combination (*ΔftsH1-4)*. Samples were taken after overnight growth in LB at 28°C. Several isolates were tested since despite the presence of the suppressor, the *ΔftsH* strain grows slowlier and tends to pick up additional mutations.
(TIF)

S6 Fig Mutations in *rdcA*/*rdcB* are not phenotypically additive with deletions of specific domains of DgcE. Macrocolonies of the *E*. *coli* K-12 strains AR3110 and the indicated mutant derivatives were grown on Congo red plates for 5 d at 28°C. All combinations of mutations tested produce a phenotype similar to that of *dgcE* or *rdcA*/*rdcB* null mutants.
(TIF)

S7 Fig The presence or absence of RdcA/RdcB has no influence on proteolytic turnover of DgcE. Immunoblot analysis was performed with a derivative of strain W3110 expressing the chromosomally encoded C-terminally 3xFLAG-tagged DgcE and the indicated mutant derivatives. Samples were taken after overnight growth in LB at 28°C.
(TIF)

S8 Fig Introducing the T103D amino acid exchange does not affect cellular levels of RdcA. **A:** Immunoblot analysis was performed with derivatives of strain W3110 expressing chromosomally encoded C-terminally 3xFLAG-tagged RdcA or RdcA^T103D^. Samples were taken at the indicated OD_578_ during growth in LB at 28°C. 'wt' indicates strain W3110 not expressing any 3xFLAG-tagged protein. **B:** Macrocolonies of the same strains as used in (A) were grown on Congo red plates for 5 d at 28°C.
(TIFF)

S1 Table Oligonucleotide primers used in the present study. Relevant nucleotides (e.g. restriction sites, mutations introduced or sequences specific for pKD4, pKD13, pKD45 and pSUB11) are labeled in boldface. All primer sequences are given from 5´- to 3´-ends.
(PDF)
