# Supplementary figures and images for: Genetic dissection of Escherichia coli's master diguanylate cyclase DgcE: Role of the N-terminal MASE1 domain and direct signal input from a GTPase partner system
Source: PLoS Genet. 2019 Apr 25;15(4):e1008059. doi: 10.1371/journal.pgen.1008059 (PMC6510439; doi:10.1371/journal.pgen.1008059)

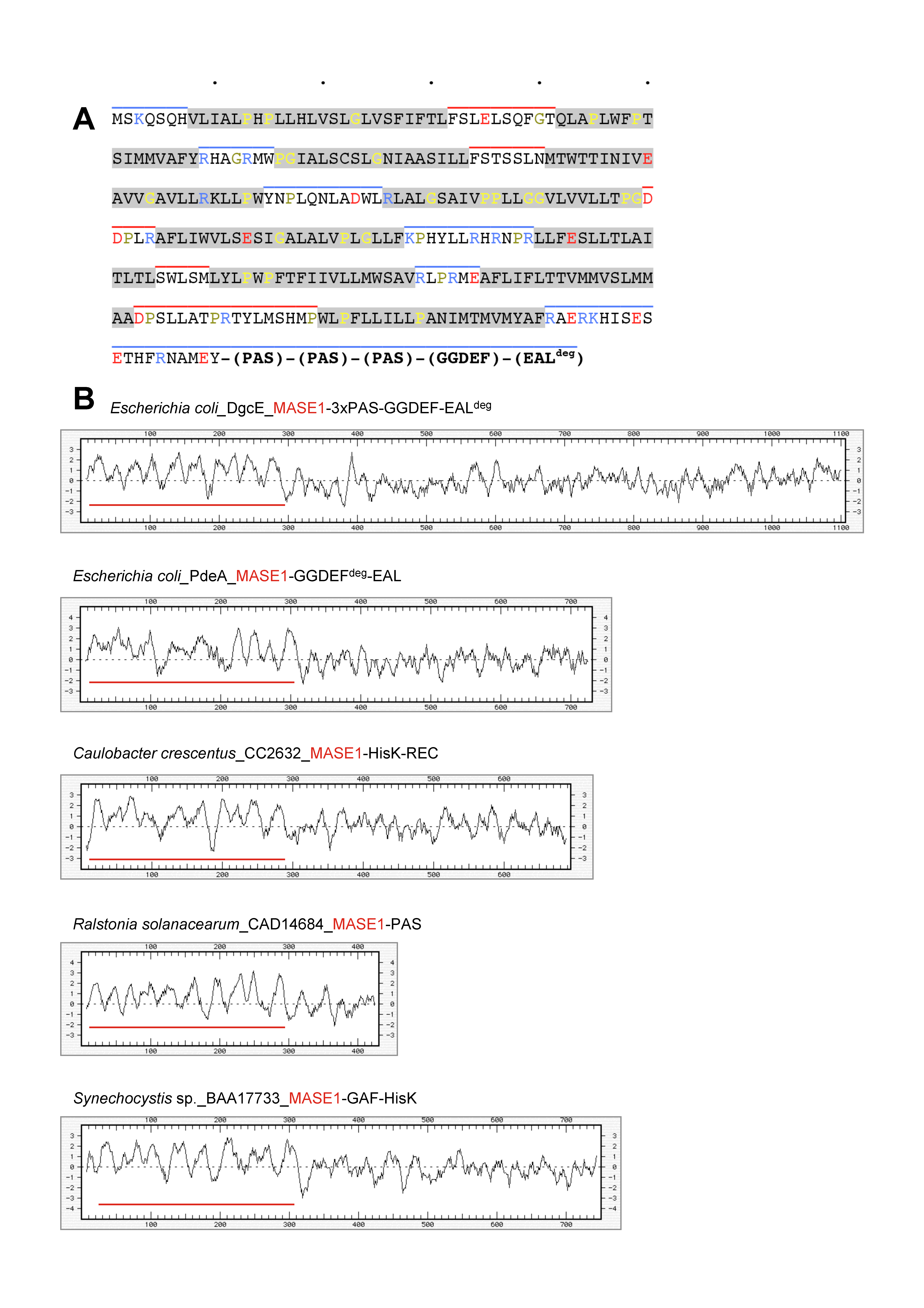

Supplement: S1 Fig — A: Sequence of the MASE1 domain of DgcE. Hydrophobic transmembrane segments are highlighted in grey, hydrophilic loop regions on the cytoplasmic and periplasmic sides of the inner membrane are overlined in blue and red, respectively. Color code of amino acids: blue, positively charged side chains; red: negatively charged side chains, dark yellow: the helix-breaking amino acids proline and glycine. B: Hydropathy plots for five proteins with N-terminal MASE1 domains that were selected to represent different clades of bacteria (E. coli: γ-proteobacteria; C. crescentus: α-proteobacteria; R. solanacearum: β-proteobacteria; Synechocystis: cyanobacteria). In all cases the MASE1 domain (highlighted in red) is discernable as a predominantly hydrophobic domain of approximately 290–300 amino acids with a characteristic hydrophobicity pattern indicative of ten transmembrane segments. A window length of 11 amino acids was used for generating the hydropathy plots [83]. (TIFF) [file pgen.1008059.s001.tiff]

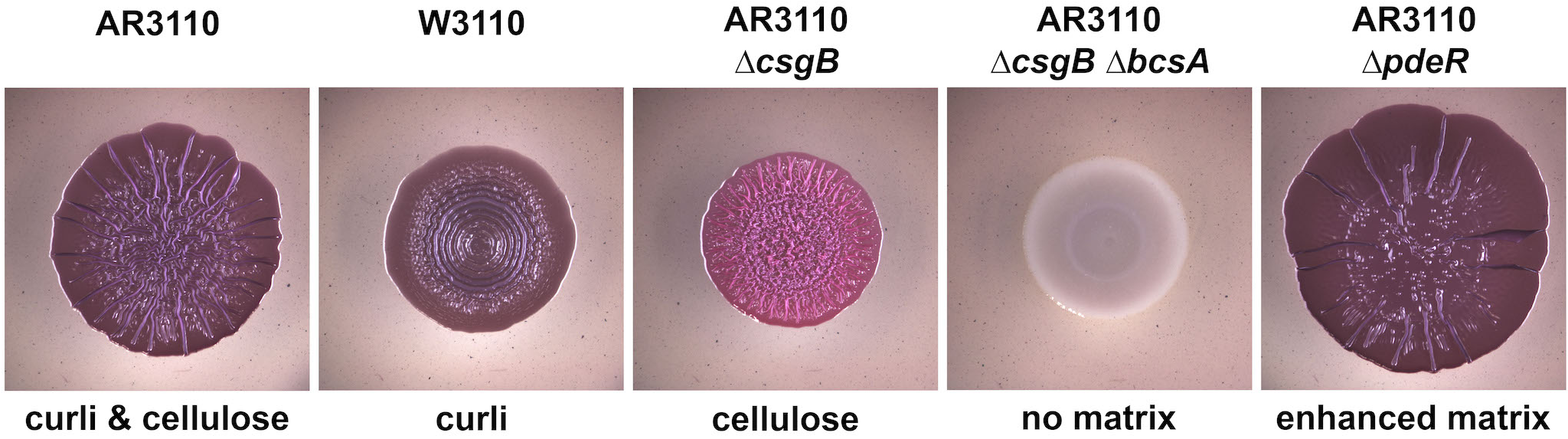

Supplement: S2 Fig — Macrocolonies of the E. coli K-12 strains AR3110 and the indicated mutant derivatives, which produce either curli or cellulose or no matrix component at all, were grown on Congo red plates for 5 d at 28°C. The pdeR knockout mutation produces higher levels of both matrix components, which results in even larger, flatter and stiffer macrocolonies, which buckle up in fewer but higher radial ridges. (TIF) [file pgen.1008059.s002.tif]

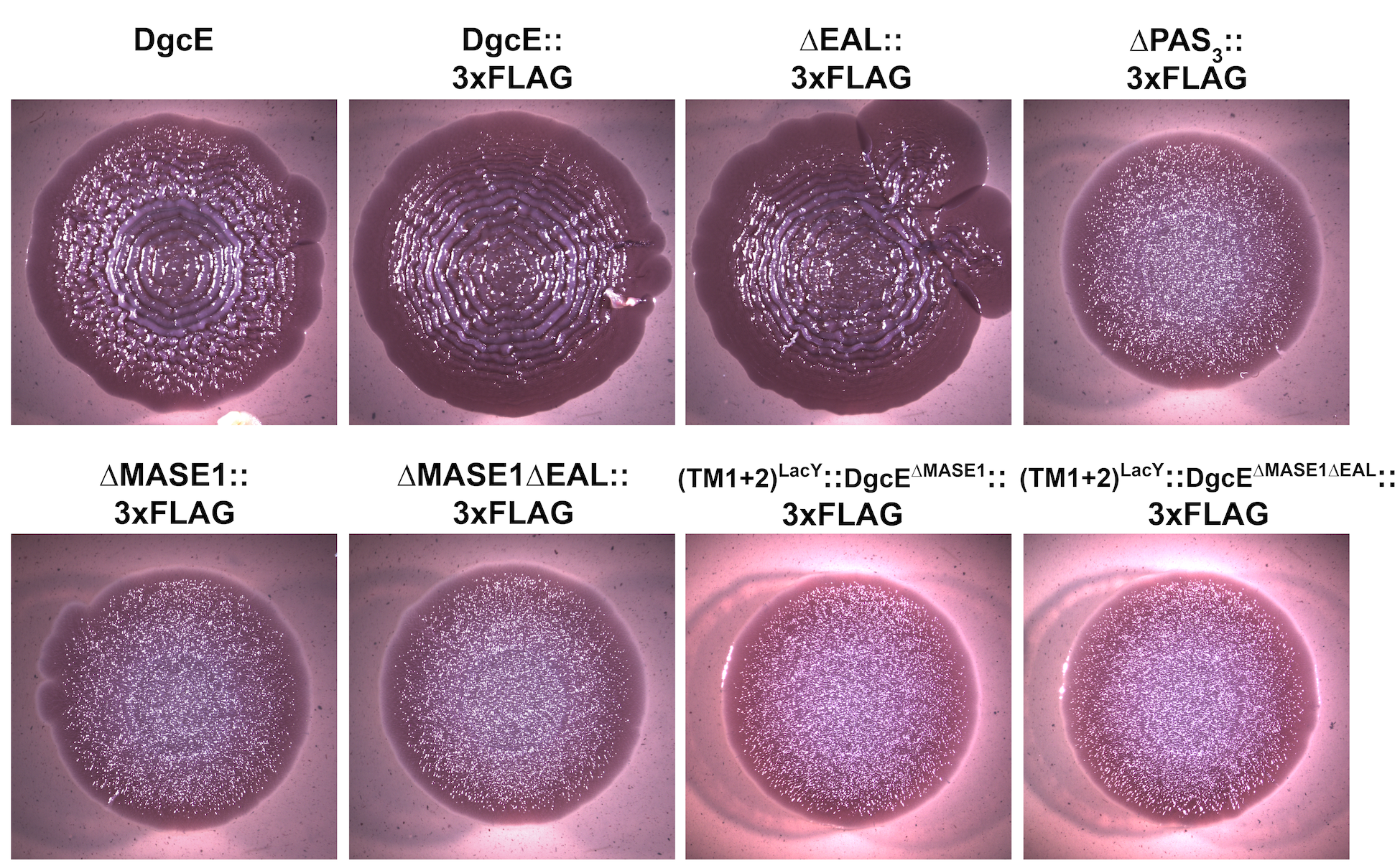

Supplement: S3 Fig — Macrocolonies of the E. coli K-12 strains W3110, which produce curli fibres but no pEtN cellulose, and the indicated chromosomal dgcE mutant derivatives (with the Flag tag sequence inserted at the 3'-end of dgcE) were grown on Congo red plates for 5 d at 28°C. (TIF) [file pgen.1008059.s003.tif]

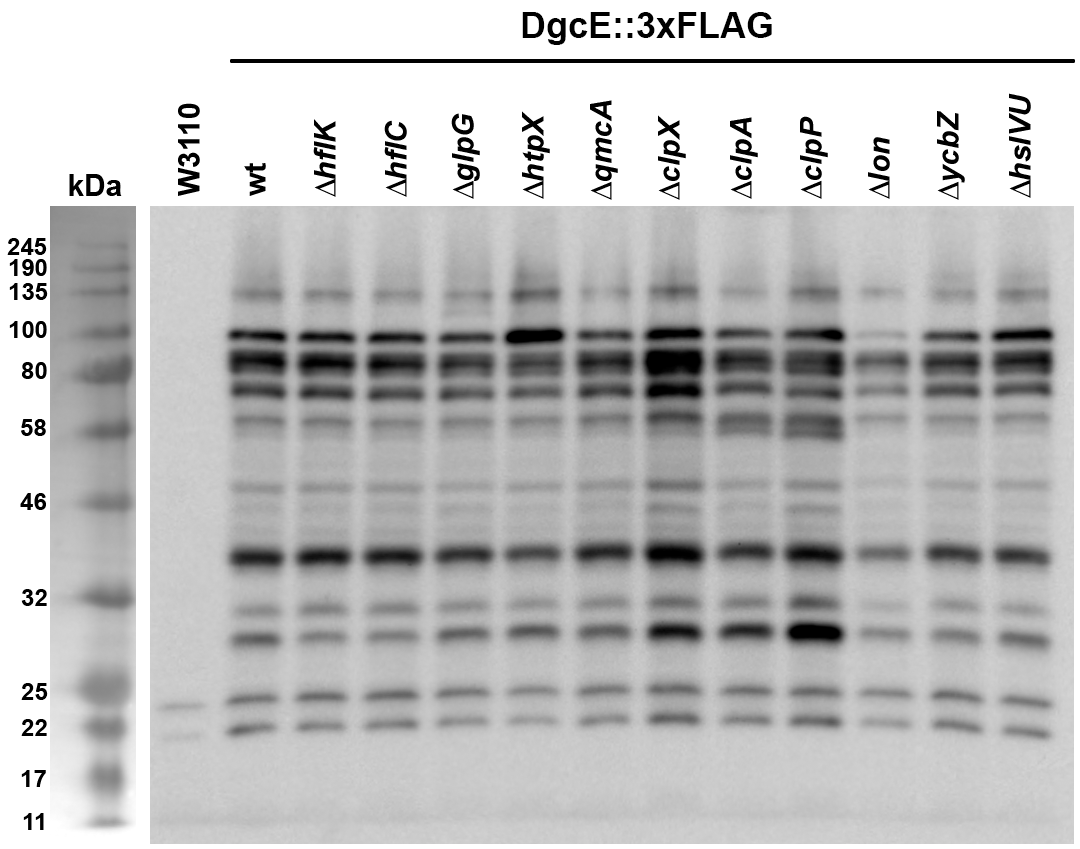

Supplement: S4 Fig — Immunoblot analysis of chromosomally encoded C-terminally 3xFLAG-tagged DgcE was performed with derivatives of strain W3110 carrying the indicated chromosomal deletion mutations, with samples taken after overnight growth in LB at 28°C. (TIF) [file pgen.1008059.s004.tif]

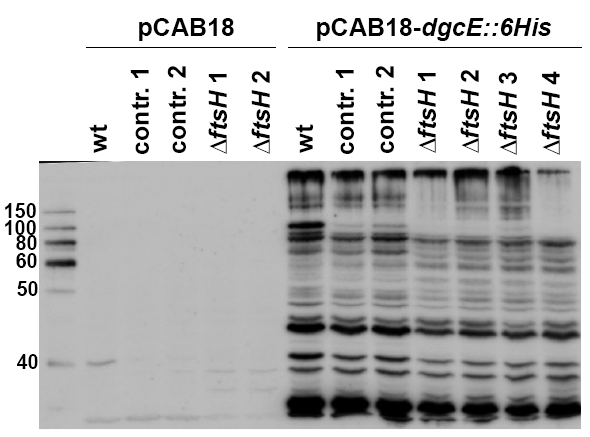

Supplement: S5 Fig — Viability of a ftsH mutant requires the presence of a specific suppressor [35]. Immunoblot analysis of plasmid-encoded C-terminally 6His-tagged DgcE was performed with the strain carrying the suppressor alone (contr. 1 and 2) or the ΔftsH and suppressor mutations in combination (ΔftsH1-4). Samples were taken after overnight growth in LB at 28°C. Several isolates were tested since despite the presence of the suppressor, the ΔftsH strain grows slowlier and tends to pick up additional mutations. (TIF) [file pgen.1008059.s005.tif]

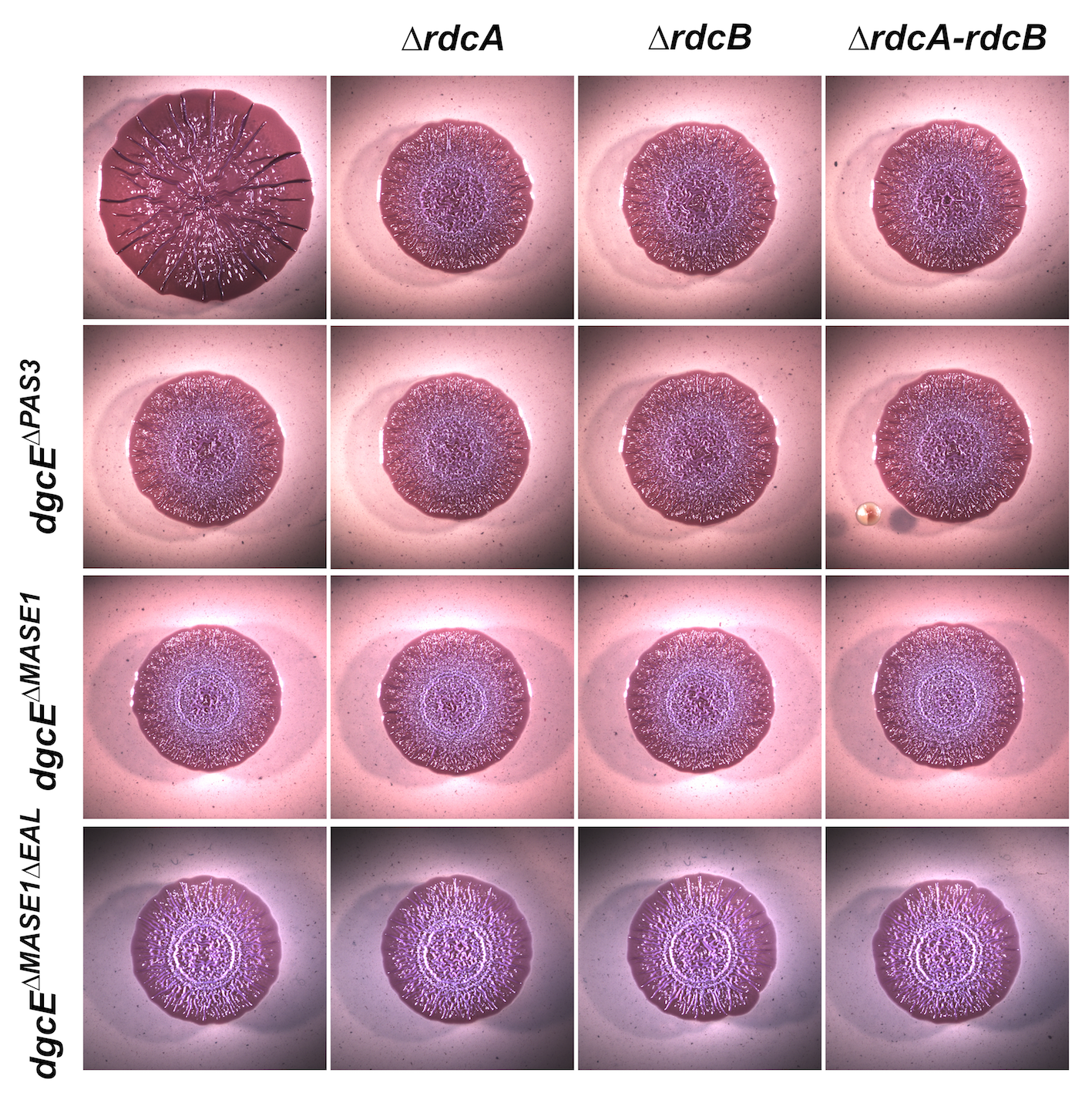

Supplement: S6 Fig — Macrocolonies of the E. coli K-12 strains AR3110 and the indicated mutant derivatives were grown on Congo red plates for 5 d at 28°C. All combinations of mutations tested produce a phenotype similar to that of dgcE or rdcA/rdcB null mutants. (TIF) [file pgen.1008059.s006.tif]

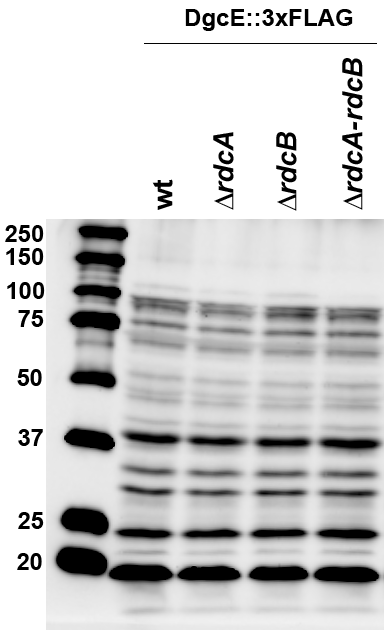

Supplement: S7 Fig — Immunoblot analysis was performed with a derivative of strain W3110 expressing the chromosomally encoded C-terminally 3xFLAG-tagged DgcE and the indicated mutant derivatives. Samples were taken after overnight growth in LB at 28°C. (TIF) [file pgen.1008059.s007.tif]

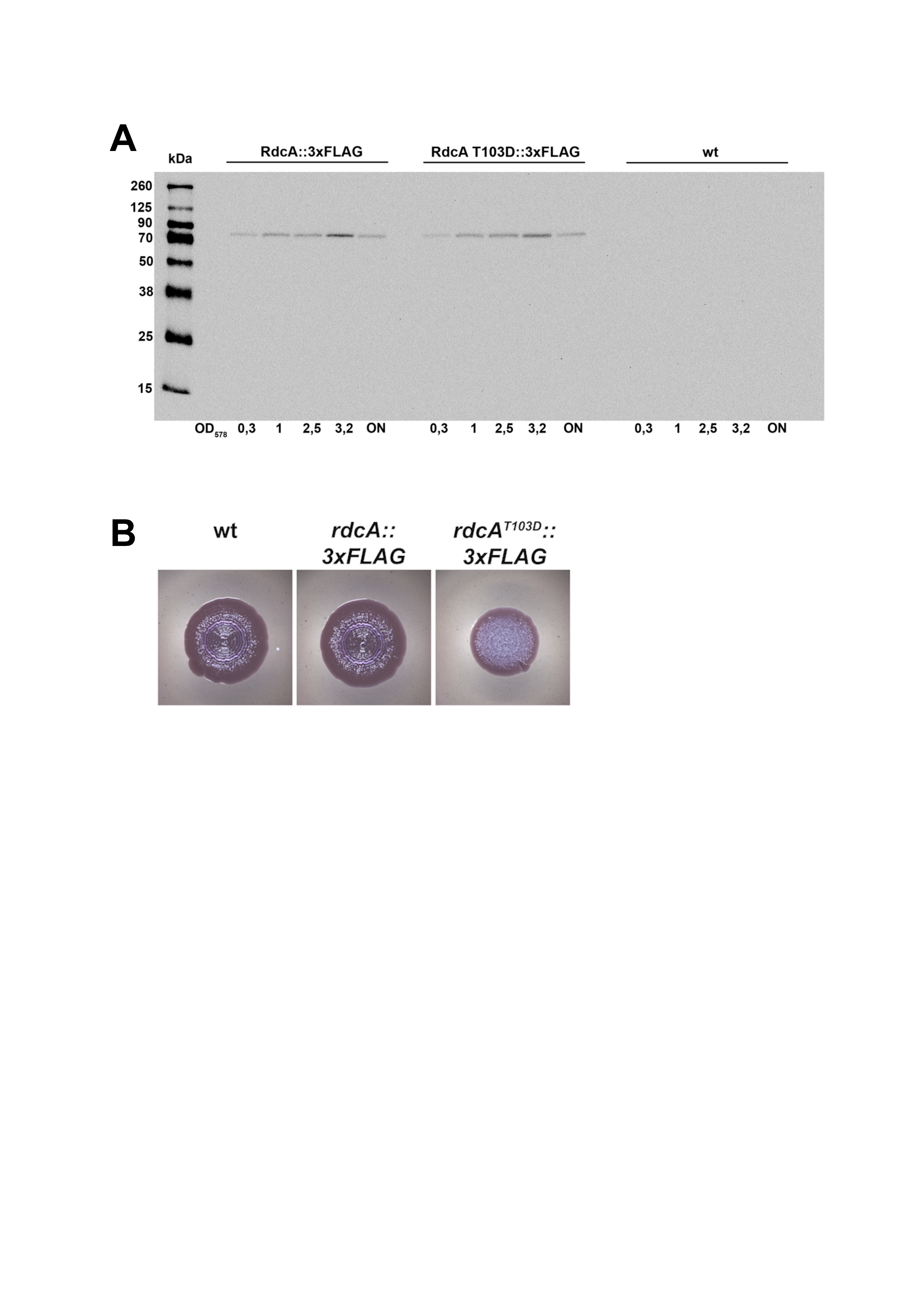

Supplement: S8 Fig — A: Immunoblot analysis was performed with derivatives of strain W3110 expressing chromosomally encoded C-terminally 3xFLAG-tagged RdcA or RdcAT103D. Samples were taken at the indicated OD578 during growth in LB at 28°C. 'wt' indicates strain W3110 not expressing any 3xFLAG-tagged protein. B: Macrocolonies of the same strains as used in (A) were grown on Congo red plates for 5 d at 28°C. (TIFF) [file pgen.1008059.s008.tiff]
