# Supplementary material for: Genetic dissection of Escherichia coli's master diguanylate cyclase DgcE: Role of the N-terminal MASE1 domain and direct signal input from a GTPase partner system
Source: PLoS Genet. 2019 Apr 25;15(4):e1008059. doi: 10.1371/journal.pgen.1008059 (PMC6510439; doi:10.1371/journal.pgen.1008059)
Supplement: S1 Table — Relevant nucleotides (e.g. restriction sites, mutations introduced or sequences specific for pKD4, pKD13, pKD45 and pSUB11) are labeled in boldface. All primer sequences are given from 5´- to 3´-ends. (PDF) [file pgen.1008059.s009.pdf]

S1 Table

Oligonucleotide primers used in the present study[1].

I. Primers used for generating chromosomal knockout mutations by one-step inactivation (OSI):

glpG-d-H1P1	5´-GCGTAACGCTTTATACTGTCCCCTTTGTGTGGAATAAGCGACAGCAACG**GTGTAGGCTGGAGCTGCTTC**-3´
glpG-u-H2P4	5´-TTATAAATCCCTGGAATTATTTTCGTTTTCGCGCATTGAGCGAATCAACA**ATTCCGGGGATCCGTCGACC**-3´
hflC-d-H1P1	5´-CGACTACCAGCGTCAGGGGGAATAACGATGCGTAAGTCAGTTATCGCGATTATC**GTGTAGGCTGGAGCTGCTTC**-3´
hflC-u-H2P4	5´-GACATCCCTGAGGATGCGGTGGCTTTATTGACCTGTACCGCAGTCGTTATA**ATTCCGGGGATCCGTCGACC**-3´
hflK-d-H1P1	5´-GGCGTCGAAAGACAACAGGGATCACCGCATAACAAATATGGAGCACAAAC**GTGTAGGCTGGAGCTGCTTC**-3´
hflK-u-H2P4	5´-CTGGTGGACGACGTGTTGCTTGCTCCACTGGTTGTGGAGGAAGAGGCTGGA**ATTCCGGGGATCCGTCGACC**-3´
hslVU-H1P1	5’-GCTCGTGACAACTATAGTAAGCGTACGCCGT**GTGTAGGCTGGAGCTGCTTC**-3’
hslVU-H2P2	5’-CGCGATTATAGGATAAAACGGCTCAGATCT**CATATGAATATCCTCCTTAG**-3’
htpX-d-H1P1	5´-GATGTGGGTATCGCATATTGCGTTTTGTTAAACTGAGGTAAAAAGAAAATT**GTGTAGGCTGGAGCTGCTTC**-3´
htpX-u-H2P4	5´-CATAAAAAAAGCGCGTCGATCAGGACGCGCTTTTTAGTATTTACTTCATA**ATTCCGGGGATCCGTCGACC**-3´
qmcA-d-H1P1	5´-TCAAGCAGCAATTTTGCTGATTATGGACGGTACAACAGGAGGTTTTTCCG**GTGTAGGCTGGAGCTGCTTC**-3´
qmcA-u-H2P4	5´-GTGGATGAACGACCATTAACTCCATCATGGCTGAGTCCGTTTGTTGGCGCA**ATTCCGGGGATCCGTCGACC**-3´
rdcA-d-H1P1	5´-CTGGCTAAATAAAATAACAAAATTTGCTTTAAGGAAGAATTTTC**GTGTAGGCTGGAGCTGCTT**-3´
rdcA-u-H2P2	5´-CCGACTCCAGCACGCGACCGGGGCCGTCAAGTAACGTCTTGGTCAA**ATGAATATCCTCCTTAG**-3´
rdcB-d-H1P1	5´-GCGATGATATTCAAACACTTTTCACGGCAGAACGATATTGACCAAG**GTGTAGGCTGGAGCTGCTTC**-3´
rdcB-u-H2P4	5´-CACACATGGATTTCGTTTAGGACTCATTGATGTAACTGATTAT**ATTCCGGGGATCCGTCGACC**-3´
rdcA-rdcB-d-H1P1	5´-GGCTAAATAAAATAACAAAATTTGCTTTAAGGAAGAATTTTCTATGTAC**GTGTAGGCTGGAGCTGCTTC**-3´
rdcA-rdcB-u-H2P4	5´-GAACGATAAAACAGCGCCCAGGCAACGACCAGGCGTTATACGCCCCA**ATTCCGGGGATCCGTCGACC**-3´
ycbZ-d-H1P1	5´-CCGGTTACGGTATAATCGCGGCTTTGACAACAGACTAAAAAACATCAAC**TGTGTAGGCTGGAGCTGCTTC**-3´
ycbZ-u-H2P4	5´-TTTATTGAAGCACGCAGGATAGCTAACACGTGTACGCTGAACAAGTCCGA**ATTCCGGGGATCCGTCGACC**-3´

II. Primers & synthetic DNA fragments used for chromosomal deletions of single domains and point mutations by two-step mutagenesis:

**(step 1)** *dgcE::ccdB*	*dgcE*(-52)-d-H1P1 (pKD45)	5´-GCATAAACACAGAAACGAATACTGGCGACCAGGTCTTGCGGATAAAGCGGTA**CGCATCGTGGCCGGATCTTGC**-3´
	dgcE(3376)-u-H2P2 (pKD45)	5´-CGATTTGTAGGCATGATAAGACGCGTTAGCGTCGCATCAGGCGATGGGGAAG**CGGATAACAGAAA**

[1] Relevant nucleotides (e.g. restriction sites, mutations introduced or pKD4, pKD13, pKD45 and pSUB11-specific sequences) labeled in **bold**. All primer sequences are given from 5´- to 3´-end.

		**GGCCGGG**-3´
**(step 2)**<br>*dgcE*$^{\Delta EAL}$	MP-d-Δ*EAL*	5´-CCTGGATGTTAGGAAGGGGGCG-3´
	MP-u-Δ*EAL*	5´-GTAGGCATGATAAGACGCGTTAGCGTCGCATCAGGCGATGGGGAAGCACGCCTCACCGTTCGCTATGTGCGGCAGCTTG-3´
**(step 2)**<br>*dgcE*$^{\Delta PAS3}$	A_*dgcE*-d-(-377)	5´-GCCGGAAGCCGATGCGCCAG-3´
	A_MP-u-Δ*PAS3*	5´-TTCGGAAATGTGTTTGCGTTCCGCAC-3´
	B_MP-d-Δ*PAS3*	5´-GTGCGGAACGCAAACACATTTCCGAAAGCGCCTCCCATGATGCACTG-3´
	B_*dgcE*-u-3516	5´-CGCCGTTATGCCGAGCGCTGG-3´

**(step 1)**<br>*TM*^{DgcE}*::ccdB*<br><br>*TM*$^{DgcE\Delta EAL}$*::ccdB*	*dgcE*(-52)-d-H1P1 (pKD45)	5´-GCATAAACACAGAAACGAATACTGGCGACCAGGTCTTGCGGATAAAGCGGTA**CGCATCGTGGCCGGATCTTGC**-3´
	dgcE(948)-u-H2P2 (pKD45)	5´-CGCCATACCGATAGCGGAATATTCCATCGCGTTCCGAAAGTGGGTTTCGCT**CGGATAACAGAAAGGCCGGG**-3´
**(step 2)**<br>*dgcE*$^{\Delta TM}$<br><br>*dgcE*$^{\Delta TM \Delta EAL}$	MP-d-Δ*TM*	5´-GCATAAACACAGAAACGAATACTGGCGACCAGGTCTTGCGGATAAAGCGGTAATGGCGGAACGCAAACACATTTCCG-3´
	MP-u-Δ*TM*	5´-GCCAGTTCGGTTTGATATGTG-3´
**(step 2)**<br>*(TM1+2)*^{LacY}*::*<br>*dgcE*$^{\Delta TM/\Delta TM\Delta EAL}$	5´-GCATAAACACAGAAACGAATACTGGCGACCAGGTCTTGCGGATAAAGCGGTAATGTACTATTTAAAAAACACAAACTTTTGGATGTTCGGTTTATTCTTTTTCTTTTACTTTTTTATCATGGGAGCCTACTTCCCGTTTTTCCCGATTTGGCTACATGACATCAACCATATCAGCAAAAGTGATACGGGTATTATTTTTGCCGCTATTTCTCTGTTCTCGCTATTATTCCAACCGCTGTTTGGTCTGCTTTCTGCGGAACGCAAACACATTTCCGAAAGCGAAACCCACTTTCGGAACGCGATGGAATATTCCGCTATCGGTATGGCG-3´	

**(step 1)**<br>*GGDEF*^{DgcE}*::ccdB*	*dgcE*(2134)-d-H1P1 (pKD45)	5´-CAGTTTTGAGAAACAACTGCGTATCCTGCTGCAAACGGTAAACAGTACACATC**CGCATCGTGGCCGGATCTTGC**-3´
	dgcE(2536)-u-H2P2 (pKD45)	5´-CTGTTCATCAAGCGACATCGCCGCCCGTTCGCTATGTGCGGCAGCTTGCTG**CGGATAACAGAAAGGCCGGG**-3´
**(step 2)**<br>*dgcE*^{GGAAF}	A_*dgcE*-d-1665	5´-GCGCCTGCACATTACGCTTG-3´
	A_MP-u-*GGAAF*	5´-CAGACCAAAT**G**CA**G**CCCCACCGAGTC-3´
	B_MP-d-*GGAAF*	5´-GACTCGGTGGGG**C**TG**C**ATTTGGTCTG-3´
	B_*dgcE*-u-2275	5´-CCGGCGGTCAAGAGCATGGC-3´

**(step 1)**<br>*rdcA(bp7-1005)::ccdB*	*rdcA(-43)*-d-H1P1 (pKD45)	5´-GGCTAAATAAAATAACAAAATTTGCTTTAAGGAAGAATTTTCTATGTAC**CGCATCGTGGCCGGATCTTGC**-3´
	rdcA-u-H2P2 (pKD45)	5´-CCGTTGTTGGCTAACTCATAGCGCGCCCGATTCGCCAGGTAGCCCCACAT**CGGATAACAGAAAGGCCGGG**-3´
**(step 2)**<br>*rdcA T103D*	A_*yjdA*-d-(-70)	5´-GCCGAAAACAGAACTCAAAAAC-3´
	A_MP-u-*yjdAT103D*	5´-GAATAAGCGTCGGCAGCGC**GTC**CATTGGGCGATTACG-3´
	B_MP-d-*yjdAT103D*	5´-CGTAATCGCCCAATG**GAC**GCGCTGCCGACGCTTATTC-3´
	B_*yjdA*-u-1108	5´-CGGCATGGGCAAAATCTTCC-3´

**(step 2)** *rdcA K82A*	A_*rdcA*-d-(-41)	5´-CTAAATAAAATAACAAAATTTGCTTTAAGGA AGAATTTTCTATGTACACACAGACCCTGTATG-3´
	A_KpnI-*rdcA-K82A*	5´-GCAATTGTC**GGTACC**ATGAAAGCAGGG**GCG** TCAACCACCATTAATGCCATTGTTGG-3´
	B_AatII-*rdcA-K82A*	5´-GGCCCG**GACGTC**CAGCGCCTTCGC-3´
	B_*yjdA*-u-1108	5´-CGGCATGGGCAAAATCTTCC-3´

III. Primers used for chromosomal C-terminal 3xFLAG-tagging of *dgcE* variants and *rdcA*:

dgcE-d-H1P1 (FLAG)	5´-GCTGGATTTGCTGGTGAATAGTAGTTATTTCGCGATTAAC**GACTACAAAG ACCATGACGG**-3´
dgcE-u-H2P2 (FLAG)	5´-CGCGTTAGCGTCGCATCAGGCGATGGGGAAGCACGCCTCA**CCTTAGTTCC TATTCCGAAGTTC**-3´
*dgcE*Δ*EAL*-d-H1P1 (FLAG)	5´-GCCGGGTGACGGTTTACGAACCGCAGCAAGCTGCCGCACATAGCGAACGG **GACTACAAAGACCATGACGG**-3´
*dgcE*Δ*EAL*-u-H2P2 (FLAG)	5´-CGCGTTAGCGTCGCATCAGGCGATGGGGAAGCACGCCTCA**CCTTAGTTCC TATTCCGAAGTTCC**-3´
rdcA-d-H1P1	5´-CCCAGCTGTTACGCGATGATATTCAAACACTTTTCACGGCAGAACGATAT **GACTACAAAGACCATGACGG**-3´
rdcA-u-H2P2	5´-GATAAACCGACTCCAGCACGCGACCGGGGCCGTCAAGTAACGTCTTGGTC A**CCTTAGTTCCTATTCCGAAGTTC**-3´

IV. Primers used for cloning into pCAB18 (Barembruch et al. 2007):

*dgcE*_EcoRI_f	5´-CGC**GAATTC**ATGAGCAAACAATCACAGC-3´
*dgcE6His*_HindIII_r	5´-GAC**AAGCTT**TCAGTGATGGTGATGGTGATGGTTAATCGCGAAATAACTAC-3´
*dgcE*_HindIII_r	5´-GAC**AAGCTT**TCAGTTAATCGCGAAATAACTAC-3´
*dgcE*Δ*EAL6His*_ HindIII_*r*	5´-GAC**AAGCTT**TCAGTGATGGTGATGGTGATGCCGTTCGCTATGTGCGGCAG-3´
*dgcE-EAL6His*_HindIII_r	5´-GAC**AAGCTT**TCAGTGATGGTGATGGTGATGGTTAATCGCGAAATAACTAC-3´
*dgcE*Δ*TM*_EcoRI-_f	5´-CGC**GAATT**CATGAGCAAAGCGGAACGCAAACACATTTCC-3´
*(TM1+2)LacY_on_dgcE*Δ*TM*_EcoRI_f	5´-CGC**GAATT**CATGAGCAAATACTATTTAAAAAACACAAAC-3´
*rdcA*_EcoRI_f	5´-CGC**GAATTC**ATGTACACACAGACCCTGTATG-3´
*rdcB*_HindIII_r	5´-GAC**AAGCTT**TTACGCCAAATTTACAGAGAAG-3´

V. Primers used for constructing bacterial two-hybrid plasmids on the basis of the pKT25, pKNT25, pUT18C and pUT18 vectors (Karimova et al. 1998):

dgcE *dgcE*$^{\Delta TM}$ *dgcE*^{EAL}	*dgcE*_XbaI_f	5´-GCC**TCTAGA**GAGCAAACAATCACAGCATG-3´
	*dgcE*Δ*TM*_XbaI_f	5´-GCC**TCTAGA**GCGTGCGGAACGCAAACACATTT CCG-3´
	*dgcE-EAL*_XbaI_f	5´-GCC**TCTAGA**GGCACATAGCGAACGGGCGGC-3´
	*dgcE*_KpnI_r	5´-GCC**GGTACC**CGGTTAATCGCGAAATAACTAC-3´
dgcE$^{\Delta EAL}$ *dgcE*^{TM}	*dgcE*_XbaI_f	5´-GCC**TCTAGA**GAGCAAACAATCACAGCATG-3´
	*dgcE*Δ*EAL*_KpnI_r	5´-GCC**GGTACC**CGCCGTTCGCTATGTGCGGCAGC TTG-3´
	*dgcE-TM*_KpnI_r	5´-GCC**GGTACC**CGTTCGGAAATGTGTTTGCGTT-3´
dgcE$^{\Delta GGDEF}$	A_*dgcE*_XbaI_f	5´-GCC**TCTAGA**GAGCAAACAATCACAGCATG-3´
	A_MP_*dgcE*-u-2133_r	5´-ATGTGTACTGTTTACCGTTTGCAGCAGGATAC-3´
	B_MP_*dgcE*Δ*GGDEF*_f	5´-GTATCCTGCTGCAAACGGTAAACAGTACACAT CAGCAAGCTGCCGCACATAGC-3´

	B_*dgcE*_KpnI_r	5´-GCC**GGTACC**CGGTTAATCGCGAAATAACTAC-3´
$PAS3^{dgcE}$<br>$GGDEF^{dgcE}$	*dgcE-PAS*$_3$_XbaI_f	5´-GCC**TCTAGA**GCACATTTCCGAAAGCGAAACC-3´
	*dgcE-GGDEF*_XbaI_f	5´-GCC**TCTAGA**GAGTACACATCAGCGACATGCC-3´
	dgcE-PAS$_3$_KpnI_r	5´-GCC**GGTACC**CGCAGTTGTTTCTCAAAACTGGC-3´
	*dgcE-GGDEF*_KpnI_r	5´-GCC**GGTACC**CGAGCTTGCTGCGGTTCGTAAAC-3´
rdcA	*rdcA*_XbaI_f	5´-GCC**TCTAGA**GTACACACAGACCCTGTATGAG-3´
	*rdcA*_BamHI_r	5´-GCC**GGATCC**CGATATCGTTCTGCCGTGAAAAG-3´
rdcB	*rdcB*_XbaI_f	5´-GCC**TCTAGA**GACCAAGACGTTACTTGACGGC-3´
	*rdcB*_KpnI_r	5´-GCC**GGTACC**CGCGCCAAATTTACAGAGAAGA-3´
